# Safety and Efficacy of Solitaire Stent Thrombectomy: Individual Patient Data Meta-Analysis of Randomized Trials

**DOI:** 10.1161/STROKEAHA.115.012360

**Published:** 2016-02-22

**Authors:** Bruce C.V. Campbell, Michael D. Hill, Marta Rubiera, Bijoy K. Menon, Andrew Demchuk, Geoffrey A. Donnan, Daniel Roy, John Thornton, Laura Dorado, Alain Bonafe, Elad I. Levy, Hans-Christoph Diener, María Hernández-Pérez, Vitor Mendes Pereira, Jordi Blasco, Helena Quesada, Jeremy Rempel, Reza Jahan, Stephen M. Davis, Bruce C. Stouch, Peter J. Mitchell, Tudor G. Jovin, Jeffrey L. Saver, Mayank Goyal

**Affiliations:** From the Department of Medicine and Neurology, Melbourne Brain Centre at the Royal Melbourne Hospital, University of Melbourne, Parkville, Australia (B.C.V.C., S.M.D.); Department of Clinical Neurosciences, Hotchkiss Brain Institute, Cumming School of Medicine, University of Calgary, Foothills Hospital, Calgary AB, Canada (M.D.H., B.K.M., A.D.); Neurology Department, Hospital Vall d’Hebron, Barcelona, Spain (M.R.); The Florey Institute of Neuroscience and Mental Health, University of Melbourne, Parkville, Australia (G.A.D.); Department of Radiology, CHUM-Hopital Notre Dame, University of Montreal, Montreal, Canada (D.R.); Department of Radiology, Beaumont Hospital, Dublin, Ireland (J.T.); Department of Neuroscience, Hospital Germans Trias i Pujol, Universitat Autònoma de Barcelona, Barcelona, Spain (L.D., M.H.-P.); Department of Neuroradiology, Hôpital Gui-de Chauliac, Montpellier, France (A.B.); Department of Neurosurgery, State University of New York at Buffalo, Buffalo, New York (E.I.L.); Department of Neurology, University Hospital of University Duisburg–Essen, Essen, Germany (H.-C.D.); Division of Neuroradiology and Division of Neurosurgery, Departments of Medical Imaging and Surgery, Toronto Western Hospital, University Health Network, University of Toronto, Toronto, Canada (V.M.P.); Department of Radiology, Hospital Clínic, Barcelona, Spain (J.B.); Department of Neurology, Hospital de Bellvitge, Barcelona, Spain (H.Q.); Department of Radiology, University of Alberta, Edmonton, Canada (J.R.); Division of Interventional Neuroradiology, Department of Radiology and Neurosurgery, David Geffen School of Medicine, University of California, Los Angeles (UCLA) (R.J.); Department of Biostatistics and Clinical Epidemiology, The Philadelphia College of Osteopathic Medicine, PA (B.C.S.); Department of Radiology, Royal Melbourne Hospital, University of Melbourne, Parkville, Australia (P.J.M.); Stroke Institute, Department of Neurology, University of Pittsburgh Medical Center (T.G.J.); Department of Neurology and Comprehensive Stroke Center, David Geffen School of Medicine at the University of California, Los Angeles (J.L.S.); and Department of Radiology, University of Calgary, Foothills Hospital, Calgary AB, Canada (M.G.).

**Keywords:** endovascular treatment, intra-arterial therapy, ischemic stroke, mechanical thrombectomy, meta-analysis, randomized controlled trial, stent retriever device, thrombolysis

## Abstract

Recent positive randomized trials of endovascular therapy for ischemic stroke used predominantly stent retrievers. We pooled data to investigate the efficacy and safety of stent thrombectomy using the Solitaire device in anterior circulation ischemic stroke.

**Methods—:**

Patient-level data were pooled from trials in which the Solitaire was the only or the predominant device used in a prespecified meta-analysis (SEER Collaboration): Solitaire FR With the Intention for Thrombectomy as Primary Endovascular Treatment (SWIFT PRIME), Endovascular Treatment for Small Core and Anterior Circulation Proximal Occlusion With Emphasis on Minimizing CT to Recanalization Times (ESCAPE), Extending the Time for Thrombolysis in Emergency Neurological Deficits—Intra-Arterial (EXTEND-IA), and Randomized Trial of Revascularization With Solitaire FR Device Versus Best Medical Therapy in the Treatment of Acute Stroke Due to Anterior Circulation Large Vessel Occlusion Presenting Within Eight Hours of Symptom Onset (REVASCAT). The primary outcome was ordinal analysis of modified Rankin Score at 90 days. The primary analysis included all patients in the 4 trials with 2 sensitivity analyses: (1) excluding patients in whom Solitaire was not the first device used and (2) including the 3 Solitaire-only trials (excluding ESCAPE). Secondary outcomes included functional independence (modified Rankin Score 0–2), symptomatic intracerebral hemorrhage, and mortality.

**Results—:**

The primary analysis included 787 patients: 401 randomized to endovascular thrombectomy and 386 to standard care, and 82.6% received intravenous thrombolysis. The common odds ratio for modified Rankin Score improvement was 2.7 (2.0–3.5) with no heterogeneity in effect by age, sex, baseline stroke severity, extent of computed tomography changes, site of occlusion, or pretreatment with alteplase. The number needed to treat to reduce disability was 2.5 and for an extra patient to achieve independent outcome was 4.25 (3.29–5.99). Successful revascularization occurred in 77% treated with Solitaire device. The rate of symptomatic intracerebral hemorrhage and overall mortality did not differ between treatment groups.

**Conclusions—:**

Solitaire thrombectomy for large vessel ischemic stroke was safe and highly effective with substantially reduced disability. Benefits were consistent in all prespecified subgroups.

## Abstract 2

Supplemental Digital Content is available in the text.

The management of ischemic stroke because of large vessel occlusion has been transformed by the publication of 5 positive randomized trials which predominantly used stent retrievers.^1–5^ These trials have led to highest-level guideline recommendations in the United States,^6^ Europe,^7^ and Canada^8^ supporting mechanical stent thrombectomy within 6 hours of ischemic stroke onset for patients with large vessel stroke because of internal carotid and middle cerebral artery occlusions.

Although each trial was positive in its own right and no major subgroup heterogeneity was observed in the individual trials, the power to detect subgroup effects was low and precision of effect size measures was limited. Further, there was variation in the device and procedural approach used in the trials. Multiple study-level meta-analyses of summary trial data have been published.^9–11^ However, individual pooled patient data meta-analysis, similar to that performed for intravenous thrombolysis, adds power, improves precision, and allows accurate interrogation of subgroups.^12^

The trialists have agreed to pool individual patient data to address these outstanding questions. In a separate report, data from all 5 trials is being analyzed to clarify aspects of treatment across diverse device therapies. The purpose of the current report is to examine treatment effects in patients treated specifically with the most common device used in the pivotal trials, the Solitaire stent retriever (Medtronic, Dublin, Ireland).

## Methods

For this report specifically analyzing the Solitaire device, studies were eligible for the primary analysis if they met the following selection criteria: (1) randomized trial of endovascular thrombectomy added to best medical therapy versus best medical therapy alone, with the Solitaire device used first in all or a majority of the interventions and (2) imaging confirmation of large vessel occlusion before study entry. Four trials met these criteria and were included in the primary analysis (SEER Collaboration): Solitaire FR With the Intention for Thrombectomy as Primary Endovascular Treatment (SWIFT PRIME), Endovascular Treatment for Small Core and Anterior Circulation Proximal Occlusion With Emphasis on Minimizing CT to Recanalization Times (ESCAPE), Extending the Time for Thrombolysis in Emergency Neurological Deficits—Intra-Arterial (EXTEND-IA), and Randomized Trial of Revascularization With Solitaire FR Device Versus Best Medical Therapy in the Treatment of Acute Stroke Due to Anterior Circulation Large Vessel Occlusion Presenting Within Eight Hours of Symptom Onset (REVASCAT). The Multicenter Randomized Clinical Trial of Endovascular Treatment for Acute Ischemic Stroke in the Netherlands (MR CLEAN) trial was not included because the Solitaire device was used in only a minority of the interventions (but is included in a separately reported, larger analysis not focused on the Solitaire device).

Data from each trial were collated by an independent statistical center which performed analyses according to a prespecified statistical analysis plan (available in the online-only Data Supplement). Commonalities and differences in trial characteristics are summarized in Table I in the online-only Data Supplement. The primary analysis included all patients enrolled in all 4 trials. Two sensitivity analyses were performed: (1) including in the endovascular arm only those patients in whom the first device actually used was Solitaire or would have been Solitaire had a target clot been still present and accessible (Solitaire intention to treat analysis) and (2) including only patients from the 3 trials that universally used Solitaire in the endovascular arm (SWIFT PRIME, EXTEND-IA, and REVASCAT). The primary outcome was degree of disability as assessed on the modified Rankin scale (mRS) at 90 days.

Prespecified subgroup analyses were age (<70 years of age versus ≥70 years and <80 years of age versus ≥80 years), sex (male/female), stroke severity (National Institutes of Health Stroke Scale [NIHSS] ≤15, 16–20, and ≥21), site of intracranial vascular occlusion (internal carotid artery, M1 and M2 middle cerebral artery), presence of tandem cervical carotid occlusion (yes/no), extent of initial early ischemic changes (Alberta Stroke Program Early CT Score [ASPECTS] 0–5, 6–8, and 9–10), administration of alteplase (yes/no), and time from onset to randomization (<5 h and ≥5 h). Onset to randomization dichotomization at 5 h was chosen to approximate the subgroup who could have endovascular treatment commenced within 6 h of onset. In addition, patients treated with alteplase within 3 hours of stroke onset (FDA label for alteplase) were examined.

Prespecified secondary efficacy outcomes were independent functional outcome (mRS 0–2) at 90 days; major early neurological recovery at 24 h, defined as a reduction in NIHSS from baseline of at least 8 points or reaching 0 to 1; and the rate of successful revascularization at end of endovascular procedure defined as modified Treatment in Cerebral Ischemia (mTICI) 2b/3 representing restoration of blood flow to >50% of the affected territory. For this analysis, final revascularization in ESCAPE patients was reclassified so that all trials used the mTICI scale which demarcates 2b as 50% to 99% restoration of blood flow to the affected territory.^13^

Safety outcomes examined were symptomatic intracerebral hemorrhage (as defined by the source trial, see Table I in the online-only Data Supplement) and mortality. The rate of radiologically defined parenchymal hematoma was also reported.

The technical efficacy and safety of the Solitaire device was also assessed in all patients in the 4 trials in which Solitaire was actually used as the first device deployed. This as-treated population did not include patients randomized to the endovascular arm who did not receive a device either because they had already reperfused by the time of catheter angiography or navigation to the target occlusion could not be accomplished.

Statistical analysis was performed by the independent statistician who merged the individual trial databases and used SAS v.9.2 (SAS Institute, Cary, NC). The primary outcome was analyzed using mixed methods ordinal logistic regression with mRS categories 5 and 6 merged and study and trial-by-treatment interaction as random effects variables. Because the trials were conducted independently, in different geographic locations and health systems, the statistical analysis plan specified random rather than fixed effects to avoid the assumption of a common effect size among these trials. Unadjusted and adjusted models were analyzed. The adjusted analysis included 7 prespecified covariates: age, sex, baseline stroke severity, site of occlusion, intravenous alteplase treatment, ASPECTS score, and time from onset to randomization. Number needed to treat (NNT) values reflecting concurrent transitions across multiple mRS levels were derived by calculating the geometric mean of the NNT values yielded by the algorithmic joint outcome table method and the permutation test method (combining mRS categories 5 and 6).^14,15^ The secondary dichotomous outcomes were analyzed using binary logistic regression with the same covariates and study and trial-by-treatment interaction as random effects variables. NNT for dichotomous outcomes was calculated as 100/absolute risk reduction. Assessment of time of reperfusion as a predictor of outcome was conducted separately in the intervention group. The adjusted probability of independent outcome in the intervention group that achieved mTICI 2b/3 reperfusion was solved using a hierarchical generalized linear mixed model with study as a random variable and onset-to-reperfusion time. Probabilities were graphed as a function of time with the probability of independent outcome regressed against time using simple linear regression to produce an estimate of effect size for each unit of time delay to treatment.

## Results

### Characteristics of the Patients

In total, the primary analytic population included 787 anterior circulation ischemic stroke patients, 401 randomized to stent thrombectomy and 386 to standard care. Of these, 650/787 (82.6%) received intravenous thrombolysis (Table 1). In the first sensitivity analysis, the Solitaire-first intention to treat, the population included 713 patients, 327 randomized to thrombectomy and 386 to standard care. In the second sensitivity analysis, of the 3 Solitaire-only trials, there were 472 patients, including 236 randomized to endovascular intervention and 236 to standard care (Tables II and III in the online-only Data Supplement).

**Table 1.**
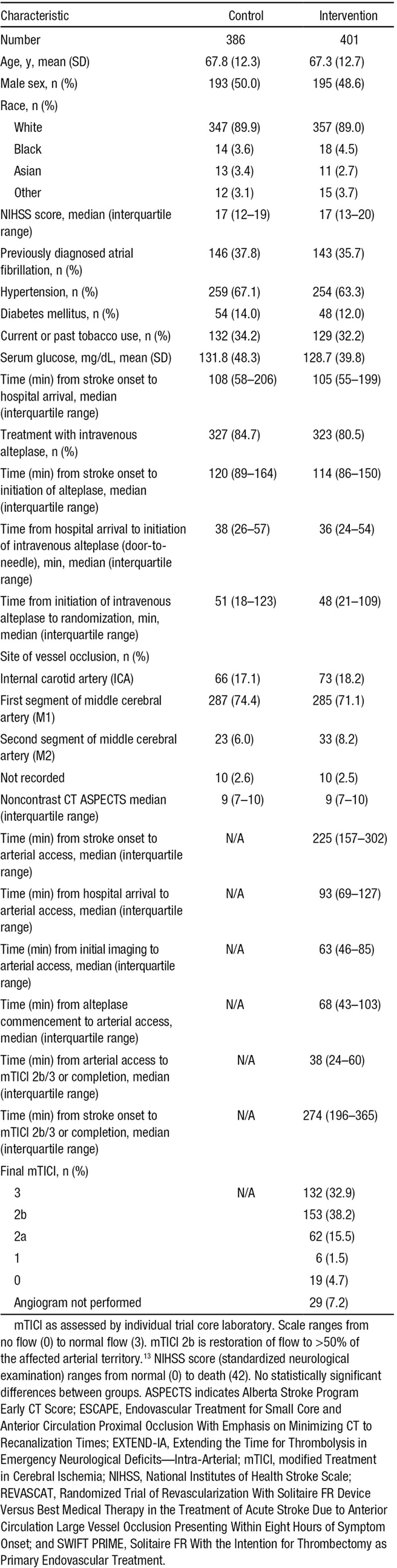
Patient and Procedural Characteristics for the Four Trials: SWIFT PRIME, ESCAPE, EXTEND-IA, and REVASCAT

### Primary Outcome

**Figure 1.**
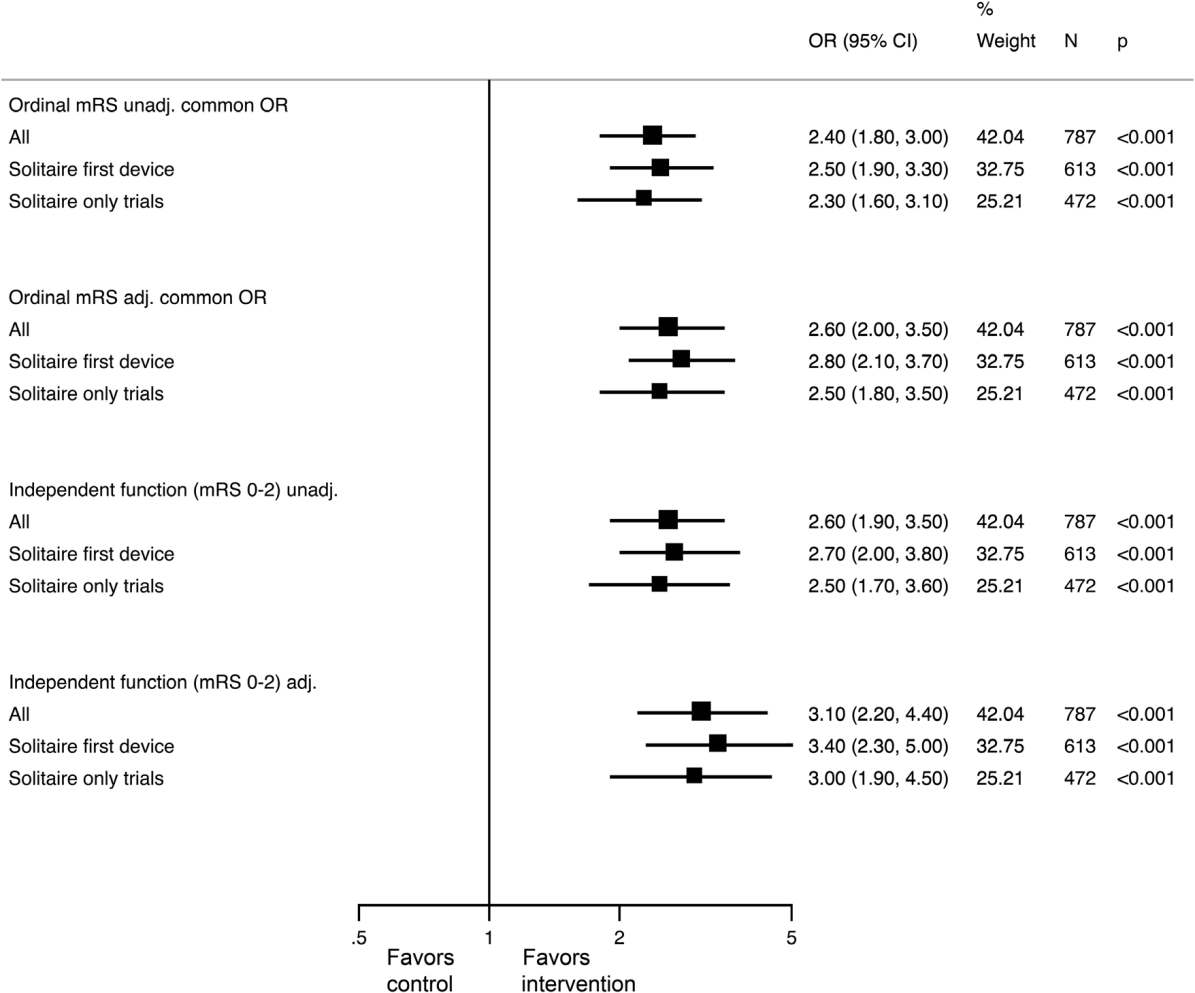
Functional outcome (modified Rankin Scale [mRS] at 90 days) in the primary and sensitivity analysis populations. Odds ratios (OR) and 95% confidence intervals (CI) for ordinal analysis of mRS (both unadjusted and adjusted for age, sex, baseline stroke severity, site of occlusion, intravenous alteplase treatment, Alberta Stroke Program Early CT Score (ASPECTS), and time from onset to randomization) and for independent functional outcome (mRS 0–2), both unadjusted and adjusted.

**Figure 2.**
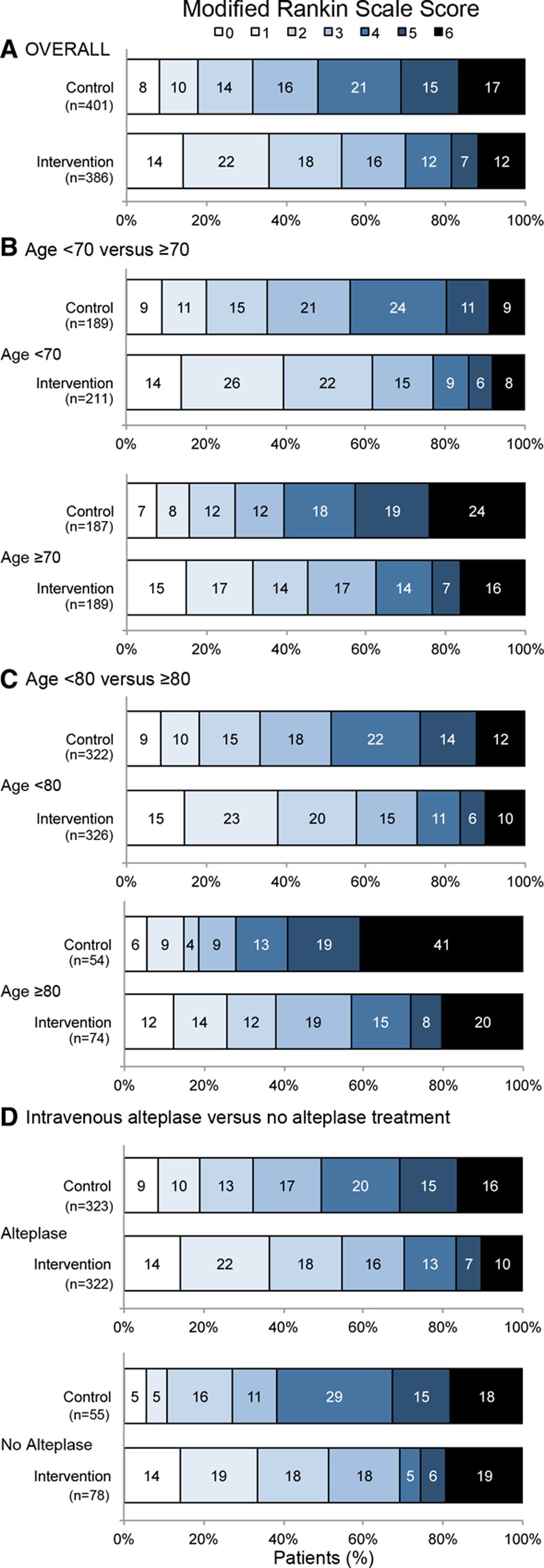
Distribution of modified Rankin scores (mRS) at 90 days in the primary analysis: SWIFT PRIME, EXTEND-IA, ESCAPE, and REVASCAT. Overall results (**A**) comparing age dichotomized at 70 years (**B**), comparing age dichotomized at 80 years (**C**), comparing those who did or did not receive intravenous alteplase before endovascular stent thrombectomy (**D**). NB mRS 5 and 6 were combined for the ordinal analysis. ESCAPE indicates Endovascular Treatment for Small Core and Anterior Circulation Proximal Occlusion With Emphasis on Minimizing CT to Recanalization Times; EXTEND-IA, Extending the Time for Thrombolysis in Emergency Neurological Deficits—Intra-Arterial; REVASCAT, Randomized Trial of Revascularization With Solitaire FR Device Versus Best Medical Therapy in the Treatment of Acute Stroke Due to Anterior Circulation Large Vessel Occlusion Presenting Within Eight Hours of Symptom Onset; and SWIFT PRIME, Solitaire FR With the Intention for Thrombectomy as Primary Endovascular Treatment.

**Figure 3.**
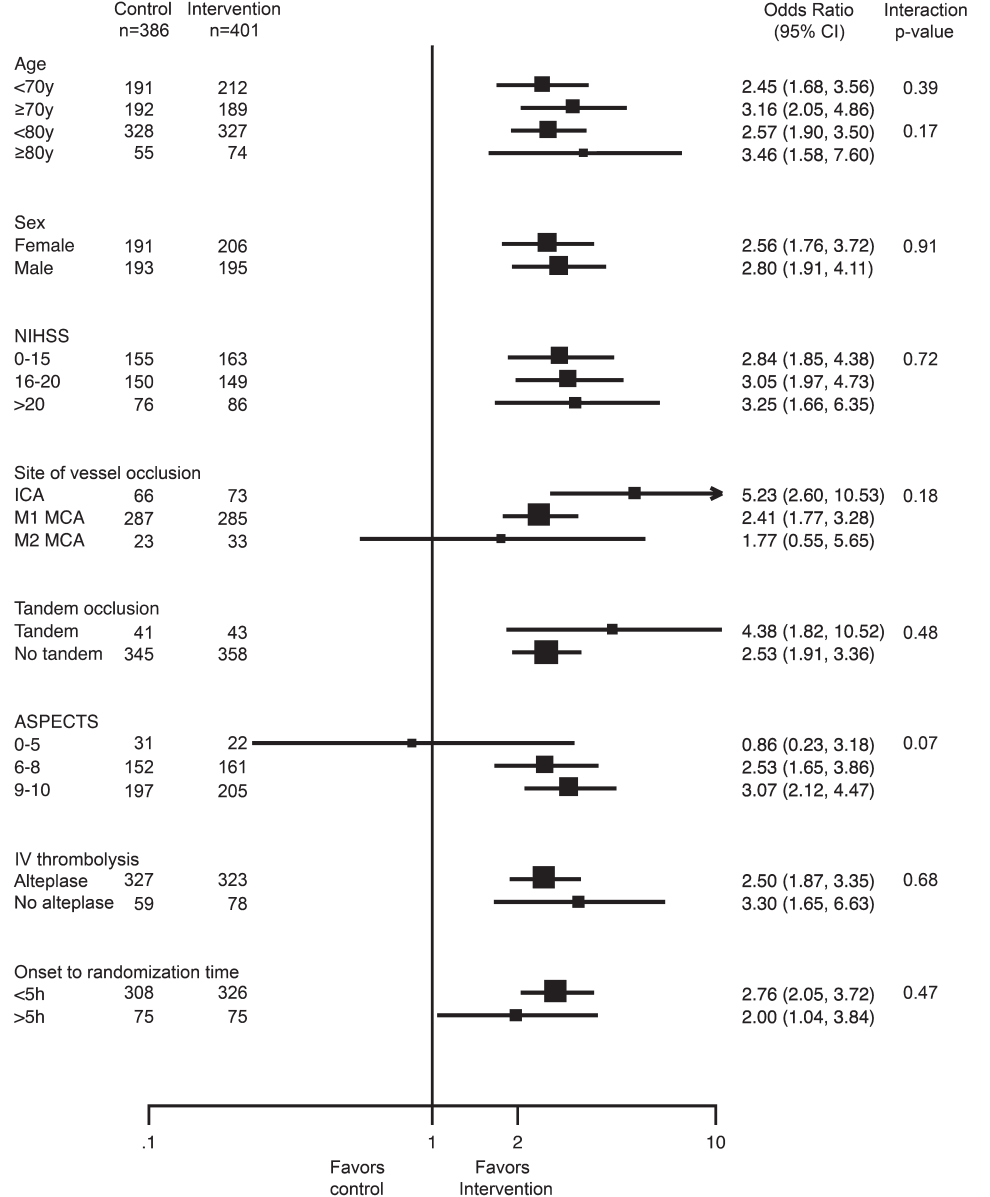
Treatment effect in predefined subgroups (Forest plot), analyses adjusted for age, sex, baseline stroke severity, site of occlusion, intravenous alteplase treatment, tandem cervical carotid occlusion, Alberta Stroke Program Early CT Score (ASPECTS), and time from onset to randomization. CI indicates confidence interval; ICA, internal carotid artery; MCA, middle cerebral artery; and NIHSS, National Institutes of Health Stroke Scale.

In the primary analysis, the common odds ratio (OR) for improvement in ordinal analysis of mRS was 2.4 (1.8–3.0; *P*=0.0000000001) unadjusted and common OR 2.7 (2.0–3.5; *P*<0.0000000001) adjusted—an NNT of 2.5 patients to improve at least one level on the mRS (Table 2 and Figures 1 and 2A). Effects were similar in the 2 sensitivity analyses (Figure 1; Figures I and II and Tables IV and V in the online-only Data Supplement) and in patients who received alteplase within 3 hours of stroke onset (Table VI in the online-only Data Supplement). There was no heterogeneity in effect in subgroup analysis by age, sex, baseline stroke severity, pretreatment thrombolysis, site of intracranial vascular occlusion, time from onset to randomization, or extent of initial noncontrast computed tomography abnormalities, with the exception of the Solitaire as first device population where there was heterogeneity in treatment effect by baseline ASPECTS score, *P*=0.02 (Figures 2B, 2C and 3). Findings were similar in the 2 sensitivity analysis populations (Figures III and IV in the online-only Data Supplement).

**Table 2.**
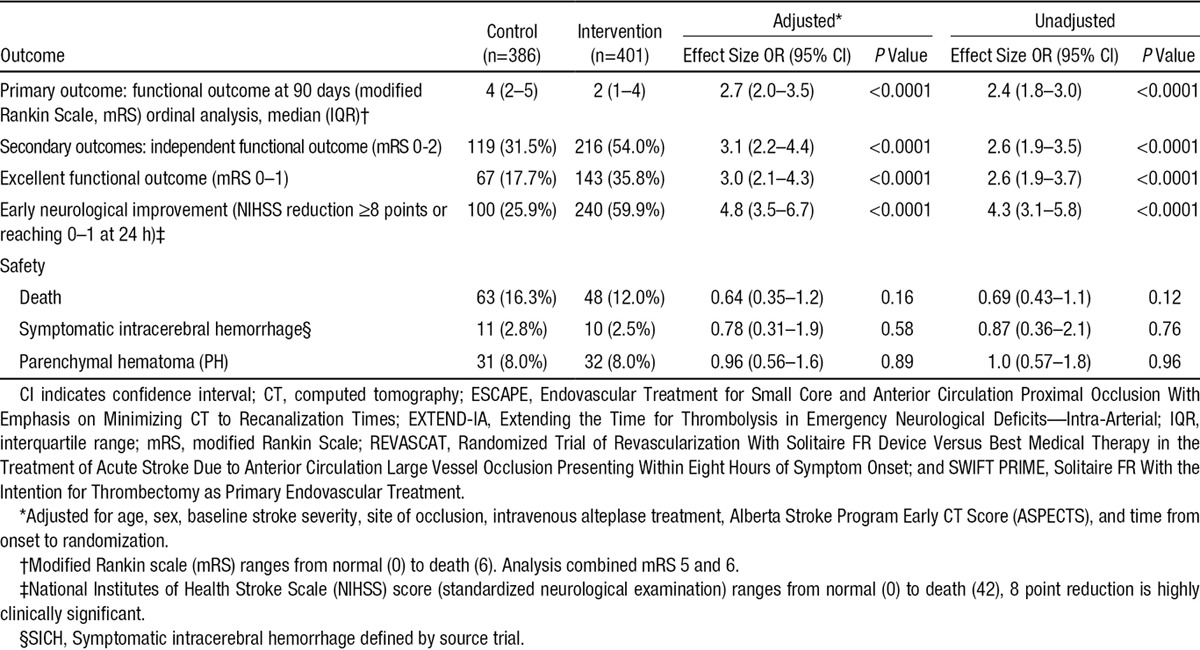
Patient Outcomes in Primary Analysis: SWIFT PRIME, ESCAPE, EXTEND-IA, REVASCAT

### Secondary Outcomes and Safety

Benefit was seen in all secondary efficacy outcomes. The NNT to achieve an extra patient with independent outcome (mRS 0–2) was 4.25 (95% confidence interval 3.29–5.99; Table 2). Major early neurological recovery was substantially increased in the Solitaire-treated patients. Findings were similar in the 2 sensitivity analysis populations (Tables IV and V in the online-only Data Supplement). In a simpler fixed effects model, there was no evidence of a study-by-treatment interaction, indicating homogeneity of effect across all 4 trials (*P*=0.513).

In the safety analyses, there were no significant differences in symptomatic hemorrhage or mortality overall (Table 2). There was, however, a significant reduction in mortality in the subgroup aged ≥80 in the complete SEER data set (20% versus 40%, adjusted OR 3.7 [1.3–10.6; *P*=0.01]; Figure 2C) with similar trend in the Solitaire sensitivity population (Figure IIIC in the online-only Data Supplement). Results were similar in those treated with alteplase within 3 hours versus 3 to 4.5 hours after stroke onset (Tables VI–VIII in the online-only Data Supplement).

**Figure 4.**
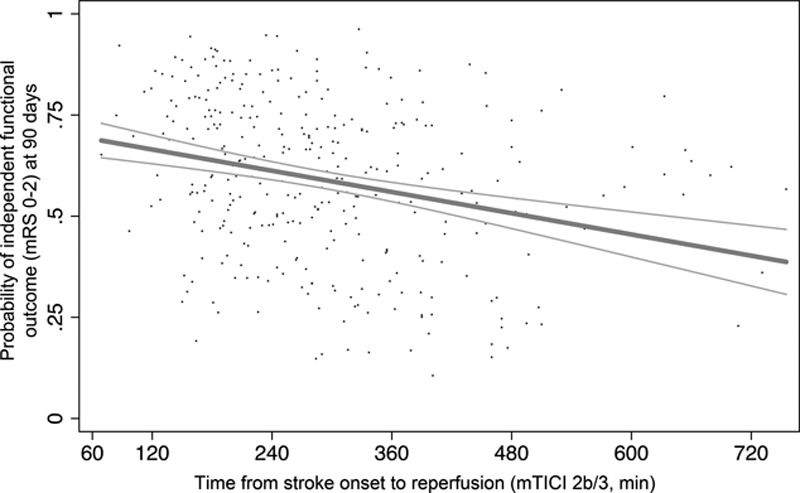
Relationship of time from stroke onset to reperfusion (modified Treatment in Cerebral Ischemia [mTICI] 2b/3) and independent functional outcome (modified Rankin scores [mRS] 0–2) with 95% confidence interval (scatter represents individual predicted outcomes in the endovascular group only). Estimates were adjusted for age, sex, baseline stroke severity on the National Institutes of Health Stroke Scale (NIHSS) score, site of occlusion, intravenous alteplase treatment, Alberta Stroke Program Early CT Score (ASPECTS), and time from onset to TICI 2b/3 flow among the patients treated with Solitaire as the first device in all 4 trials and achieving mTICI 2b/3 at end of procedure. The onset-to-TICI 2b/3 time was a significant predictor of outcome (odds ratio [OR] 0.99 per minute; *P*=0.011) with the probability of independent functional outcome declining 1% per 23 minute delay.

In the technical efficacy analysis, among patients from all 4 trials harboring persisting occlusions at catheter angiography and actually treated with Solitaire as first device used, the rate of successful revascularization (mTICI 2b/3) was 236/306 (77%). Rates of mRS 0 to 2 increased with each successive category of mTICI (*P*=0.01 for trend; Table IX in the online-only Data Supplement). There was a small but significant reduction in the proportion of Solitaire-treated patients achieving independent outcome as time from onset to reperfusion increased (Figure 4).

## Discussion

This individual patient data meta-analysis has demonstrated robust benefit of Solitaire stent thrombectomy. The degree of benefit conferred is substantial, with 40 of every 100 patients treated having reduced disability as a result of thrombectomy, including 23 patients achieving an independent outcome. No major safety concerns were noted, with no increase in symptomatic hemorrhage or mortality. Benefit was homogenous across a broad range of patients, including younger and older, male and female, internal carotid and middle cerebral artery clot locations, presence or absence of tandem cervical carotid occlusion, milder and more severe deficits, milder and more severe ischemic injury on initial imaging, and in those who received alteplase or were alteplase-ineligible.

Older age has often been used as an exclusion criterion for thrombectomy, and indeed 2 of the 4 trials analyzed had an upper age limit (SWIFT PRIME and REVASCAT). Nonetheless, in patients with good or independent premorbid function, there was no evidence of reduced treatment effect in the elderly and, moreover, a clinically and statistically significant 20% absolute reduction in mortality in patients aged ≥80 in the SEER trials. There is, therefore, no justification for exclusion from thrombectomy purely on the basis of age in clinical practice.

Initial analyses of Interventional Management of Stroke (IMS-3) and recent combined analysis with MR CLEAN focused on stroke severity (NIHSS≥20) as a key determinant of endovascular treatment benefit.^16,17^ Our analyses demonstrated at least as great a treatment benefit in those with NIHSS≤15 as in those with NIHSS>20. Although few patients were enrolled in the recent trials with NIHSS<6, there is no evidence of treatment effect modification across the available severity spectrum. Treatment of mild stroke will continue to require clinical judgment.^18^

The preponderance of patients in these trials received intravenous alteplase before endovascular thrombectomy and fibrinolytic treatment was part of the inclusion criteria for EXTEND-IA and SWIFT PRIME. All patients who were alteplase-eligible in the analyzed trials were given alteplase. These data, therefore, support the continued use of alteplase before thrombectomy in all eligible patients. Although there were fewer patients in these trials who were alteplase-ineligible, there was clear benefit of endovascular thrombectomy in these patients not candidates for pretreatment with fibrinolytic agents, confirming the benefits of endovascular thrombectomy in this group.

The crucial effect of time has been emphasized in relation to intravenous thrombolysis^12,19^ and also applies to endovascular therapies.^20,21^ In the case of alteplase, time to treatment is the most commonly analyzed metric as time of reperfusion is infrequently documented and may occur several hours post-treatment. The precise quantification of time to reperfusion and the higher frequency of reperfusion with endovascular treatment should allow more detailed understanding of the relationship of time to outcome. The proportion of patients with favorable imaging decreases over time such that earlier imaging should increase the proportion eligible for treatment and the overall beneficial effect to the stroke population.^22^ Our pooled analysis confirmed a time–benefit relationship, with decline in frequency of independent outcome with longer onset to reperfusion times. However, the effect size is small in this analysis, and it is likely that these studies underestimate the importance of time because of selective recruitment of patients with good quality collateral flow or penumbral profiles. The impact of time has previously been shown to be muted in patients with favorable imaging profiles.^23^ Accordingly, in clinical practice, it is essential to streamline systems to minimize delays and achieve optimal patient outcomes.

The Solitaire device for stent thrombectomy had an overall rate of successful revascularization (mTICI 2b/3) of 236/306 (77%) across these studies with a low rate of symptomatic hemorrhage. Although further device innovation to improve the rates of complete reperfusion (mTICI 3) on first pass of the device will undoubtedly occur, these results set a clear benchmark for future technological development.

In seeking to characterize the effects of the Solitaire stent retriever, the inclusion of trials in which other endovascular treatments were used has the potential to introduce confounds. We eliminated this concern by confining this analysis to studies that used the Solitaire device in a majority of patients and by performing sensitivity analyses confined to the patients treated with the Solitaire device.

Limitations of this study include the potential heterogeneity in inclusion criteria between studies. However, we found no evidence of a study-by-treatment interaction and analysis at the level of individual patient data minimizes the risk of bias. All of the 4 trials specified that patients were included on the basis of imaging, and treatment was conducted quickly once imaging eligibility had been ascertained. Thus, certain patient groups were not included in the trials in sufficient numbers to draw conclusions regarding efficacy. This particularly applies to those with large ischemic core, defined using ASPECTS, poor collateral grade, or unfavorable penumbral patterns. The point estimate for treatment effect was unfavorable in the small group of patients with baseline ASPECTS 0 to 5. However, benefit was not statistically excluded and may accrue in some of these patients, depending on infarct volume, location, and patient comorbidities.^24^ More advanced imaging may improve the reliability of core estimation versus noncontrast computed tomography and provide greater information about infarct topography. This analysis has focused on the endovascular trials using only or predominantly the Solitaire device and does not provide detailed evidence regarding other endovascular devices or approaches. Further individual patient data meta-analysis in a broader range of endovascular trials is planned.

This analysis confirms the robust treatment benefits of endovascular stent thrombectomy using the Solitaire device in patients with large vessel occlusion ischemic stroke, selected by imaging and treated rapidly within 6 hours of stroke onset. No clinical effect modifiers were identified, indicating that age and stroke severity (within the range included in the trials) should not exclude patients from therapy. Effects in later time windows and in patients with more extensive irreversible brain injury at baseline require further study.

## Sources of Funding

Medtronic provided an unrestricted grant to the investigators to support this analysis.

## Disclosures

Dr Campbell reports research support from the National Health and Medical Research Council of Australia (GNT1043242 and GNT1035688), Royal Australasian College of Physicians, Royal Melbourne Hospital Foundation, National Heart Foundation, and National Stroke Foundation of Australia and unrestricted grant funding for the EXTEND-IA trial to the Florey Institute of Neuroscience and Mental Health from Covidien (Medtronic). Dr Hill reports unrestricted grant funding for the ESCAPE trial and SEER collaboration to University of Calgary from Covidien (Medtronic) and from Hoffmann-La Roche Canada Ltd for the TEMPO2 trial. Dr Menon reports honoraria from Penumbra Inc. and honorary board membership of QuikFlo Health Inc. Dr Demchuk reports unrestricted grant support for the ESCAPE trial and speaker’s fees from Medtronic. Dr Roy reports a grant from University of Calgary for angiography core laboratory activities. Dr Thornton reports acting as a scientific consultant for Neuravi. Dr Bonafe reports personal fees from Covidien (Medtronic). Dr Levy reports personal fees from Covidien (Medtronic) and Abbott and has acted as a consultant for Stryker, NeXtGen Biologics, Abbott Vascular, and Pulsar Vascular. He holds stock in Intratech Medical Ltd, Blockade Medical LLC, and Medina Medical Inc. In addition, Dr Levy renders expert legal opinion in his expertise as a neurosurgeon for attorneys. Dr Diener reports honoraria from Medtronic and advisory board membership for Medtronic. Dr Pereira reports personal fees from Covidien (Medtronic). Dr Jahan has served as consultant for Medtronic. Dr Davis reports lecture fees from Covidien (Medtronic), Boehringer Ingelheim, and Bristol-Myers Squibb and has served on an advisory board for Boehringer Ingelheim. Dr Mitchell has served as an unpaid advisory board member for Medtronic. Dr Jovin has consulted for Codman Neurovascular and Neuravi, holds stock in Silk Road and Blockade, has acted as an unpaid consultant to Stryker as PI of the DAWN trial, and served as an unpaid member of a Medtronic Advisory Board. Dr Saver is an employee of the University of California. Dr Saver has served as an unpaid site investigator in multicenter trials run by Medtronic and Stryker for which the UC Regents received payments on the basis of clinical trial contracts for the number of subjects enrolled. Dr Saver received stock options for services as a scientific consultant regarding trial design and conduct to Cognition Medical. Dr Saver receives funding for services as a scientific consultant regarding trial design and conduct to Medtronic/Covidien, Stryker, Neuravi, BrainsGate, Pfizer, Squibb, Boehringer Ingelheim (prevention only), ZZ Biotech, and St Jude Medical. Dr Saver serves as an unpaid consultant to Genentech advising on the design and conduct of the PRISMS trial; neither the University of California nor Dr Saver received any payments for this voluntary service. The University of California has released the Rankin Focused Assessment for free use under a Creative Commons u license and has copyright for Rankin Scale training vignettes. The University of California has patent rights in retrieval devices for stroke. Dr Goyal reports unrestricted funding for the ESCAPE trial from Covidien (Medtronic), speaker’s honoraria from Medtronic and Stryker, and has acted as a consultant for Microvention. The other authors report no conflicts.

## Supplementary Material
