# Supplementary material for: Safety and Efficacy of Solitaire Stent Thrombectomy: Individual Patient Data Meta-Analysis of Randomized Trials
Source: Stroke. 2016 Feb 22;47(3):798–806. doi: 10.1161/STROKEAHA.115.012360 (PMC4760381; doi:10.1161/STROKEAHA.115.012360)
Supplement: Supplementary file 1 [file str-47-798-s001.pdf]

SUPPLEMENTAL MATERIAL

Individual patient data meta-analysis of randomized trials of Solitaire stent thrombectomy

Bruce C.V. Campbell MBBS PhD, Michael D. Hill MD MSc, Marta Rubiera MD, Bijoy K. Menon MD MSc, Andrew Demchuk MD, Geoffrey A. Donnan MD, Daniel Roy MD, John Thornton MD, Laura Dorado MD PhD, Alain Bonafe MD, Elad I. Levy, MD, Hans-Christoph Diener MD PhD, María Hernández-Pérez MD PhD, Vitor M. Pereira MD, Jordi Blasco MD, Helena Quesada MD, Jeremy Rempel MD, Reza Jahan MD, Stephen M. Davis MD, Bruce C. Stouch PhD, Peter J. Mitchell MBBS, Tudor G. Jovin MD, Jeffrey L. Saver MD, Mayank Goyal MD for the SEER collaborators.

Index:

Table I – Summary of individual trial characteristics

Table II-III – Patient and procedural characteristics for sensitivity analyses

Table IV-V – Patient outcomes in the sensitivity analyses

Tables VI-VIII – Patient outcomes for those treated with alteplase within 0-3 hours and 3-4.5 hours after stroke onset

Table IX – Rates of mTICI at final angiogram and associated rates of independent outcome

Figures I-II – Distribution of modified Rankin scores at 90 days in the sensitivity analyses

Figures III-IV – Treatment effect in pre-defined subgroups (Forest plots) for sensitivity analyses

Statistical Analysis Plan

Table I – Summary of individual trial characteristics

Trial	number	Onset to arterial access window	Age limits	Mean age enrolled	NIHSS limits	Median NIHSS enrolled	Proportion treated with alteplase	Device	Vessel occlusion site	Imaging selection	General Anaesthesia	Onset to arterial access (median, min)	Successful revascularization (mTICI 2b/3)	Definition of SICH
ESCAPE	316	12 (84%<6 h)	≥18	71.0	≥6	17	72%	Any approved (79% stent retriever, 61% Solitaire)	ICA/M1/M1 equivalent (all M2s)	CT+CTA/+/-mCTA collateral scoring +/-CTP	9%	200	76%*	Any ICH judged to cause ≥2 point increase NIHSS
EXTEND-IA	70	6	≥18	69.4	No limits	15	100%	Solitaire	ICA/M1/M2	CT+CTA +CTP	36%	210	86%	PH2/SAH + ≥4 point increase NIHSS
SWIFT PRIME	196	6	18-80/85†	65.7	8-30	17	100%	Solitaire	ICA/M1	CT+CTA +/-CTP or MRI DWI+MRA+/-PWI	37%	224	88%	Any PH/SAH/IVH + ≥4 point increase NIHSS
REVASCAT	206	8 (90%<6 h)	18-80/85‡	66.5	≥6	17	73%	Solitaire	ICA/M1	CT+CTA +/-CTP	7%	269	66%	PH2 + ≥4 point increase NIHSS

NIHSS – National Institutes of Health Stroke Scale, **mTICI** – modified Treatment in Cerebral Ischaemia grading of angiographic reperfusion (2b is >50% reperfusion of the affected territory), **ICA** – internal carotid artery, **MCA** – middle cerebral artery: M1 first segment, M2 second segment.
CT – non-contrast computer tomography, **CTA** – CT angiography, mCTA – multiphase CT angiography for collateral scoring, **CTP** – CT perfusion, MRI – magnetic resonance imaging, **MRA** – magnetic resonance angiography.
* ESCAPE reported 72% revascularization using Thrombolysis in Cerebral Infarction (TICI) scale where 2b is >66% reperfusion of the affected territory – this was re-scored defining mTICI 2b>50% for this meta-analysis.
† SWIFT PRIME upper limit age 85 at trial start; after 1st 72 patients amended to upper limit age 80.
‡ REVASCAT amended protocol to include 80-85 year olds if CT ASPECTS>8

Table II – Patient and procedural characteristics for sensitivity analysis 1: SWIFT PRIME, EXTEND-IA, REVASCAT and ESCAPE (excluding those in whom a device other than Solitaire was used first)

Characteristic	Control	Intervention
Number	386	327
Age, yr, mean (SD)	67.8 (12.3)	66.8 (12.4)
Male sex – no. (%)	193 (50.0)	156 (47.7)
Race – no. (%)<br>White<br>Black<br>Asian<br>Other	 347 (89.9)<br>14 (3.6)<br>13 (3.4)<br>12 (3.1)	 295 (90.2)<br>13 (4.0)<br>5 (1.5)<br>14 (4.3)
NIHSS score, Median (Inter-quartile range)	17 (12-19)	17 (13-19)
Previously diagnosed atrial fibrillation – no. (%)	146 (37.8)	114 (34.9)
Hypertension – no. (%)	259 (67.1)	203 (62.1)
Diabetes mellitus – no. (%)	54 (14.0)	43 (13.1)
Current or past tobacco use – no. (%)	132 (34.2)	114 (34.9)
Serum glucose – mg/dL – mean (sd)	131.8 (48.3)	128.2 (39.3)
Time (min) from stroke onset to hospital arrival, Median (Inter-quartile range)	108 (58-206)	108 (57-197)
Treatment with intravenous alteplase – no. (%)	327 (84.7)	266 (81.3)
Time (min) from stroke onset to initiation of alteplase, Median (Inter-quartile range)	120 (89-164)	117 (90-155)
Time from hospital arrival to initiation of intravenous alteplase (door-to-needle) – min<br>Median (Inter-quartile range)	38 (26-57)	39 (25-55)
Time from initiation of intravenous alteplase to randomization – min<br>Median (Inter-quartile range)	51 (18-123)	50 (22-112)
Site of vessel occlusion – no. (%)<br>Internal carotid artery (ICA)<br>First segment of middle cerebral artery (M1)<br>Second segment of middle cerebral artery (M2)<br>Not recorded	 66 (17.1)<br>287 (74.4)<br>23 (6.0)<br>10 (2.6)	 52 (15.9)<br>237 (72.5)<br>30 (9.2)<br>8 (2.4)
Non-contrast CT ASPECTS<br>– Median (Inter-quartile range)	9 (7-10)	9 (7-10)
Time (min) from stroke onset to arterial access, median (IQR)	N/A	235 (164-301)

Time (min) from hospital arrival to arterial access, median (IQR)	N/A	98 (72-131)
Time (min) from initial imaging to arterial access, median (IQR)	N/A	65 (47-86)
Time (min) from alteplase commencement to arterial access, median (IQR)	N/A	68 (45-102)
Time (min) from arterial access to TICI 2b/3 or completion, median (IQR)	N/A	38 (25-61)
Time (min) from stroke onset to mTICI 2b/3 or completion, median (IQR)	N/A	280 (201-366)
Final mTICI – no. (%)*		
3	N/A	119 (36.4)
2b		117 (35.8)
2a		49 (15.0)
1		5 (1.5)
0		16 (4.9)
Angiogram not performed		21 (6.4)

NIHSS - National Institutes of Health Stroke Scale (NIHSS) score (standardized neurological examination) ranges from normal (0) to death (42)
No statistically significant differences between groups

* mTICI: modified Treatment in Cerebral Ischemia classification, as assessed by individual trial core laboratory, i.e. TICI 2b >50%. Scale ranges from no flow (0) to normal flow (3).[7]

Table III – Patient and procedural characteristics for sensitivity analysis 2: SWIFT PRIME, EXTEND-IA and REVASCAT (Solitaire only trials)

Characteristic	Control	Intervention
Number	236	236
Age, yr, mean (SD)	67.3 (10.6)	65.8 (12.0)
Male sex – no. (%)	122 (51.7)	116 (49.2)
Race – no. (%)<br>White<br>Black<br>Asian<br>Other	 216 (91.5)<br>8 (3.4)<br>4 (1.7)<br>8 (3.4)	 213 (90.3)<br>10 (4.2)<br>1 (0.4)<br>12 (5.1)
NIHSS score, Median (Inter-quartile range)	17.0 (12-19)	17.0 (14-20)
Previously diagnosed atrial fibrillation – no. (%)	86 (36.4)	82 (34.7)
Hypertension – no. (%)	151 (64.0)	149 (63.1)
Diabetes mellitus – no. (%)	42 (17.8)	36 (15.3)
Current or past tobacco use – no. (%)	100 (42.4)	96 (40.7)
Serum glucose – mg/dL – mean (sd)	132.5 (45.7)	129.3 (40.3)
Time (min) from stroke onset to hospital arrival,<br>Median (Inter-quartile range)	105 (61-201)	100 (57-189)
Treatment with intravenous alteplase – no. (%)	209 (88.6%)	203 (86.0%)
Time (min) from stroke onset to initiation of alteplase, Median (Inter-quartile range)	115 (90-155)	118 (90-157)
Time from hospital arrival to initiation of intravenous alteplase (door-to-needle) – min<br>Median (Inter-quartile range)	40 (31-60)	43 (29-56)
Time from initiation of intravenous alteplase to randomization – min<br>Median (Inter-quartile range)	76 (36-140)	59 (29-121)
Site of vessel occlusion – no. (%)<br>Internal carotid artery (ICA)<br>First segment of middle cerebral artery (M1)<br>Second segment of middle cerebral artery (M2)<br>Not recorded	 27 (11.4)<br>182 (77.1)<br>20 (8.5)<br>7 (3.0)	 28 (11.9)<br>174 (73.7)<br>27 (11.4)<br>7 (3.0)
Non-contrast CT ASPECTS<br>– Median (Inter-quartile range)	8 (7-10)	8 (7-10)
Time (min) from stroke onset to arterial access, median (IQR)	N/A	235 (171-290)
Time (min) from hospital arrival to arterial access, median (IQR)	N/A	104 (79-137)

Time (min) from initial imaging to arterial access, median (IQR)	N/A	75 (51-90)
Time (min) from alteplase commencement to arterial access, median (IQR)	N/A	74 (53-103)
Time (min) from arterial access to TICI 2b/3 or completion, median (IQR)	N/A	49 (29-66)
Time (min) from stroke onset to mTICI 2b/3 or completion, median (IQR)	N/A	287 (218-367)
Final mTICI – no. (%)*		
3	N/A	94 (39.8)
2b		73 (30.9)
2a		33 (14.0)
1		3 (1.3)
0		13 (5.5)
Angiogram not performed		20 (8.5)

NIHSS - National Institutes of Health Stroke Scale (NIHSS) score (standardized neurological examination) ranges from normal (0) to death (42)
No statistically significant differences between groups

* mTICI: modified Treatment in Cerebral Ischemia classification, as assessed by individual trial core laboratory, i.e. TICI 2b >50%. Scale ranges from no flow (0) to normal flow (3).[7]

Table IV – Patient outcomes in the sensitivity analysis 1: SWIFT PRIME, EXTEND-IA, REVASCAT and ESCAPE (excluding those in whom a device other than Solitaire was used first)

Outcome	Control (n=386)	Intervention (n=327)	Adjusted*		Unadjusted	
			Effect size OR (95%CI)	p value	Effect size OR (95%CI)	p value
**Primary outcome**<br>Functional outcome at 90 days (Modified Rankin Scale – mRS) Ordinal analysis† – median (IQR)	4 (2-5)	2 (1-4)	2.8 (2.1-3.7)	<0.001	2.5 (1.9-3.3)	<0.001
**Secondary Outcomes**<br>Independent functional outcome (mRS0-2)	119 (30.8)	182 (55.7)	3.4 (2.3-5.0)	<0.001	2.7 (2.0-3.8)	<0.001
Excellent functional outcome (mRS0-1)	67 (17.4)	120 (36.7)	3.2 (2.2-4.7)	<0.001	2.7 (1.9-3.9)	<0.001
Early neurological improvement (NIHSS reduction ≥8 points or reaching 0–1 at 24h)‡	100 (25.9)	195 (59.6)	4.9 (3.5-6.9)	<0.001	4.2 (3.1-5.8)	<0.001
**Safety**						
Death	63 (16.3%)	41 (12.5%)	0.71 (0.41 to 1.2)	0.23	0.72 (0.44 to 1.2)	0.17
Symptomatic intracerebral hemorrhage§	11 (2.8%)	8 (2.4%)	0.70 (0.26 to 1.9)	0.47	0.84 (0.32 to 2.2)	0.73
Parenchymal hematoma (PH)	31 (8.0%)	30 (9.2%)	1.1 (0.63 to 1.9)	0.77	1.1 (0.58 to 2.2)	0.70

* for age, sex, baseline stroke severity, site of occlusion, intravenous alteplase treatment, ASPECTS score, and time from onset to randomization

† Modified Rankin scale (mRS) ranges from normal (0) to death (6). Analysis combined mRS 5 & 6

‡ National Institutes of Health Stroke Scale (NIHSS) score (standardized neurological examination) ranges from normal (0) to death (42), 8 point reduction is highly clinically significant.

§ SICH - Symptomatic intracerebral hemorrhage defined by source trial

Table V – Patient outcomes in the sensitivity analysis 2: SWIFT PRIME, EXTEND-IA and REVASCAT (Solitaire only trials)

Outcome	Control (n=236)	Intervention (n=236)	Adjusted*		Unadjusted	
			Effect size OR (95%CI)	p value	Effect size OR (95%CI)	p value
**Primary outcome** Functional outcome at 90 days (Modified Rankin Scale – mRS) Ordinal analysis† – median (IQR)	3.0 (IQR 2-5)	2.0 (IQR 1-4)	2.5 (1.8 to 3.5)	<0.0001	2.3 (1.6 to 3.1)	<0.0001
**Secondary Outcomes** Independent functional outcome (mRS0-2)	76 (32.2%)	129 (54.7%)	3.0 (1.9 to 4.5)	<0.0001	2.5 (1.7 to 3.6)	<0.0001
Excellent functional outcome (mRS0-1)	41 (17.4%)	85 (36.0%)	2.9 (1.8 to 4.7)	<0.0001	2.7 (1.7 to 4.1)	<0.0001
Early neurological improvement (NIHSS reduction ≥8 points or reaching 0–1 at 24h)‡	61 (25.8%)	141 (59.7%)	4.9 (3.0 to 8.0)	<0.0001	4.3 (2.9 to 6.3)	<0.0001
**Safety**						
Death	35 (14.8%)	31(13.1%)	0.82 (0.42 to 1.6)	0.57	0.83 (0.46 to 0.67)	0.54
Symptomatic intracerebral hemorrhage§	7 (3.0%)	4 (1.7%)	0.47 (0.13 to 1.7)	0.26	0.44 (0.08 to 2.5)	0.35
Parenchymal hematoma (PH)	25 (10.6%)	23 (9.7%)	0.84 (0.45 to 1.6)	0.59	0.92 (0.46 to 1.8)	0.81

* for age, sex, baseline stroke severity, site of occlusion, intravenous alteplase treatment, ASPECTS score, and time from onset to randomization

† Modified Rankin scale (mRS) ranges from normal (0) to death (6). Analysis combined mRS 5 & 6

‡ National Institutes of Health Stroke Scale (NIHSS) score (standardized neurological examination) ranges from normal (0) to death (42), 8 point reduction is highly clinically significant.

§ SICH - Symptomatic intracerebral hemorrhage defined by source trial

Table VI – Patient outcomes for those treated with alteplase within 0-3 hours of stroke onset (in accordance with US FDA label) versus 3-4.5 hours after stroke onset in the primary analysis: SWIFT PRIME, EXTEND-IA, REVASCAT and ESCAPE (entire SEER dataset)

Outcome	IV alteplase only (n=264)	IV alteplase + Endovascular (n=280)	Adjusted*		Unadjusted	
			Effect size (95%CI)	p value	Effect size (95%CI)	p value
**Alteplase commenced 0-3h**						
**Primary outcome**<br>Functional outcome at 90 days (Modified Rankin Scale – mRS) Ordinal analysis† – median (IQR)	3 (2-5)	2 (1-4)	2.4 (1.8, 3.3)	<0.001	2.3 (1.7, 3.1)	<0.001
**Secondary Outcomes**<br>Independent functional outcome (mRS0-2)	88 (33.3)	158 (64	3.0 (2.0, 4.4)	<0.001	2.6 (1.8, 3.7)	<0.001
Excellent functional outcome (mRS0-1)	52 (19.7)	107 (38.2)	2.6 (1.7, 3.9)	<0.001	2.5(1.7, 3.7)	<0.001
Early neurological improvement (NIHSS reduction ≥8 points or reaching 0–1 at 24h)‡	74 (28.0)	172 (61.4)	4.7 (3.1, 7.0)	<0.001	4.1 (2.8, 5.9)	<0.001
**Safety**						
Death	43 (16.3)	28 (10.0)	0.54 (0.25-1.2)	0.12	0.57 (0.34-0.97)	0.04
Symptomatic intracerebral hemorrhage§	8 (3.0)	8 (2.9)	0.73 (0.26, 2.1)	0.56	0.92 (0.30, 2.8)	0.88
Parenchymal hematoma (PH)	24 (9.1)	16 (5.7)	0.54 (0.27, 1.1)	0.08	0.61 (0.31, 1.2)	0.14

**Alteplase commenced 3-4.5h**	N=63	N=49				
Outcome	IV alteplase only (n=264)	IV alteplase + Endovascular (n=280)	Adjusted*		Unadjusted	
			Effect size (95%CI)	p value	Effect size (95%CI)	p value
**Primary outcome**<br>Functional outcome at 90 days (Modified Rankin Scale – mRS) Ordinal analysis† – median (IQR)	4 (3-5)	3 (2-4)	3.1 (1.5, 6.5)	0.003	2.8 (1.4, 5.6)	0.004
**Secondary Outcomes**<br>Independent functional outcome (mRS0-2)	14 (22.2)	20 (40.8)	3.7 (1.3, 10.4)	0.01	2.3 (1.0, 5.3)	0.05
Excellent functional outcome (mRS0-1)	8 (12.7)	12 (24.5)	2.8 (0.93, 8.3)	0.07	2.1 (0.79, 5.8)	0.13
Early neurological improvement (NIHSS reduction ≥8 points or reaching 0–1 at 24h)‡	13 (20.6)	24 (49.0)	2.9 (1.2, 7.2)	0.02	3.7 (1.6, 8.5)	0.003
Safety						
Death	11 (7.5)	5 (10.2)	0.40 (0.10-1.5)	0.17	0.52 (0.16-1.6)	0.26
Symptomatic intracerebral hemorrhage§	2 (3.2)	1(2.0)	0.88 (0.05, 16.3)	0.93	0.64 (0.05, 7.4)	0.72
Parenchymal hematoma (PH)	6 (9.5)	9 (18.4)	1.8 (0.52, 6.4)	0.34	2.1 (0.70, 6.6)	0.18

* adjusted for age, sex, baseline stroke severity, site of occlusion, intravenous alteplase treatment, ASPECTS score, and time from onset to randomization

† Modified Rankin scale (mRS) ranges from normal (0) to death (6). Analysis combined mRS 5 & 6

‡ National Institutes of Health Stroke Scale (NIHSS) score (standardized neurological examination) ranges from normal (0) to death (42), 8 point reduction is highly clinically significant.

§ SICH - Symptomatic intracerebral hemorrhage defined by source trial

Table VII – Patient outcomes for those treated with alteplase within 0-3 hours of stroke onset (in accordance with US FDA label) versus 3-4.5 hours after stroke onset in sensitivity analysis 1: SWIFT PRIME, EXTEND-IA, REVASCAT and ESCAPE (excluding those in whom a device other than Solitaire was used first)

Outcome	IV alteplase only (n=264)	IV alteplase + Endovascular (n=229)	Adjusted*		Unadjusted	
			Effect size OR (95%CI)	p value	Effect size OR (95%CI)	p value
**Alteplase commenced 0-3h**						
**Primary outcome** Functional outcome at 90 days (Modified Rankin Scale – mRS) Ordinal analysis† – median (IQR)	4 (2-5)	2 (1-4)	2.6 (1.8, 3.9)	<0.001	2.4 (1.7, 3.3)	<0.001
**Secondary Outcomes** Independent functional outcome (mRS0-2)	88 (33.3)	134 (58.5)	3.1 (2.1, 4.7)	<0.001	2.7 (1.9, 4.0)	<0.001
Excellent functional outcome (mRS0-1)	52 (19.7)	91 (39.7)	2.8 (1.8, 4.3)	<0.001	2.6 (1.8, 4.0)	<0.001
Early neurological improvement (NIHSS reduction ≥8 points or reaching 0–1 at 24h)‡	74 (28.0)	141 (61.6)	5.0 (3.3, 7.6)	<0.001	4.1 (2.8, 6.0)	<0.001
**Safety**						
Death	43 (16.3)	24 (10.5)	0.57 (0.27, 1.2)	0.15	0.60 (0.32, 1.1)	0.11
Symptomatic intracerebral hemorrhage§	8 (3.0)	6 (2.6)	0.54 (0.14, 2.0)	0.36	0.85 (0.25, 2.8)	0.79
Parenchymal hematoma (PH)	24(9.1)	14 (6.1)	0.54 (0.26, 1.1)	0.09	0.63 (0.31, 1.25)	0.18

Alteplase commenced 3-4.5h	N=63	N=42				
Outcome	IV alteplase only (n=176)	IV alteplase + Endovascular (n=178)	Adjusted*		Unadjusted	
			Effect size OR (95%CI)	p value	Effect size OR(95%CI)	p value
**Primary outcome** Functional outcome at 90 days (Modified Rankin Scale – mRS) Ordinal analysis† – median (IQR)	4 (3-5)	3 (2-4)	3.1 (1.4, 6.7)	0.006	2.9 (1.4, 5.9)	0.005
**Secondary Outcomes** Independent functional outcome (mRS0-2)	14 (22.2)	17 (40.5)	3.9 (1.3, 11.6)	0.01	2.3 (0.96, 5.4)	0.06
Excellent functional outcome (mRS0-1)	8 (12.7)	10 (23.8)	2.5 (0.81, 7.9)	0.11	2.1 (0.73, 5.9)	0.17
Early neurological improvement (NIHSS reduction ≥8 points or reaching 0–1 at 24h)‡	13 (20.6)	20 (47.6)	2.7 (1.0, 6.9)	0.05	3.5 (1.5, 8.3)	0.005
Safety						
Death	11 (17.5)	5 (11.9)	2.1 (0.55, 8.3)	0.27	1.6 (0.51, 5.2)	0.40
Symptomatic intracerebral hemorrhage§	2 (3.2)	1 (2.4)	0.99 (0.05, 19.1)	0.99	0.76 (0.07, 9.0)	0.83
Parenchymal hematoma (PH)	6 (9.5)	9 (21.4)	2.2 (0.61, 7.6)	0.23	2.6 (0.80, 8.4)	0.11

* for age, sex, baseline stroke severity, site of occlusion, intravenous alteplase treatment, ASPECTS score, and time from onset to randomization

† Modified Rankin scale (mRS) ranges from normal (0) to death (6). Analysis combined mRS 5 & 6

‡ National Institutes of Health Stroke Scale (NIHSS) score (standardized neurological examination) ranges from normal (0) to death (42), 8 point reduction is highly clinically significant.

§ SICH - Symptomatic intracerebral hemorrhage defined by source trial

**Table VIII – ** Patient outcomes for those treated with alteplase within 0-3 hours of stroke onset (in accordance with US FDA label) versus 3-4.5 hours after stroke onset in the sensitivity analysis 2: SWIFT PRIME, EXTEND-IA and REVASCAT (3 Solitaire-only trials)

Outcome	IV alteplase only (n=176)	IV alteplase + Endovascular (n=178)	Adjusted[*]		Unadjusted	
			Effect size OR (95%CI)	p value	Effect size OR(95%CI)	p value
**Alteplase commenced 0-3h**						
**Primary outcome** Functional outcome at 90 days (Modified Rankin Scale – mRS) Ordinal analysis[†] – median (IQR)	3 (2-5)	2 (1-4)	2.3 (1.4, 3.6)	<0.001	2.3 (1.6, 3.3)	<0.001
**Secondary Outcomes** Independent functional outcome (mRS0-2)	61 (34.7)	105 (59%)	2.9 (1.8, 4.7)	<0.001	2.6 (1.7, 4.1)	<0.001
Excellent functional outcome (mRS0-1)	33 (18.8)	72 (40.4)	2.9 (1.7, 4.8)	<0.001	2.9 (1.8, 4.8)	<0.001
Early neurological improvement (NIHSS reduction ≥8 points or reaching 0–1 at 24h)[‡]	6 (16.7)	17 (54.8)	6.4 (1.5, 26.9)	0.01	4.4 (2.8, 6.9)	<0.001
**Safety**						
Death	24 (13.6)	21 (11.8)	0.87 (0.44, 1.7)	0.69	0.84 (0.45, 1.6)	0.59
Symptomatic intracerebral hemorrhage[§]	6 (3.4)	4 (2.2)	0.57 (0.15, 2.2)	0.41	0.53 (0.08, 3.6)	0.52
Parenchymal hematoma (PH)	19 (10.8)	12 (6.7)	0.47 (0.21, 1.1)	0.07	0.59 (0.28, 1.3)	0.18

**Alteplase commenced 3-4.5h**	N=36	N=31				
Outcome	IV alteplase only (n=176)	IV alteplase + Endovascular (n=178)	Adjusted*		Unadjusted	
			Effect size OR (95%CI)	p value	Effect size OR(95%CI)	p value
**Primary outcome** Functional outcome at 90 days (Modified Rankin Scale – mRS) Ordinal analysis† – median (IQR)	4 (2-5)	3 (2-4)	2.6 (0.93, 7.3)	0.07	2.5 (1.0, 6.1)	0.05
**Secondary Outcomes** Independent functional outcome (mRS0-2)	9 (25.0)	13 (41.9)	4.4 (1.0, 18.8)	0.05	2.1 (0.72, 6.0)	0.17
Excellent functional outcome (mRS0-1)	5 (13.9)	7 (22.6)	2.1 (0.48, 9.3)	0.32	1.7 (0.48, 6.3)	0.39
Early neurological improvement (NIHSS reduction ≥8 points or reaching 0–1 at 24h)‡	6 (16.7)	17 (54.8)	6.4 (1.5, 26.9)	0.01	6.1 (1.9, 19.1)	0.003
**Safety**						
Death	6 (16.7)	4 (12.9)	0.44 (0.05, 3.6)	0.44	0.69 (0.15, 3.2)	0.63
Symptomatic intracerebral hemorrhage§	1 (2.8)	0 (0)	NA	NA	NA	NA
Parenchymal hematoma (PH)	5 (13.9)	6 (19.4)	1.2 (0.26, 5.8)	0.79	1.5 (0.40, 5.6)	0.55

* for age, sex, baseline stroke severity, site of occlusion, intravenous alteplase treatment, ASPECTS score, and time from onset to randomization

† Modified Rankin scale (mRS) ranges from normal (0) to death (6). Analysis combined mRS 5 & 6

‡ National Institutes of Health Stroke Scale (NIHSS) score (standardized neurological examination) ranges from normal (0) to death (42), 8 point reduction is highly clinically significant.

§ SICH - Symptomatic intracerebral hemorrhage defined by source trial

Table IX – Rates of mTICI at final angiogram and associated rates of independent outcome in the patients treated with the Solitaire as first device used (n=306), p=0.01 for trend.

mTICI	mRS 0-2 - n (%)
0-1	8/21 (38%)
2a	25/49 (51%)
2b	62/117 (53%)
3	77/119 (65%)

Figure I – Distribution of modified Rankin scores at 90 days in the sensitivity analysis 1: SWIFT PRIME, EXTEND-IA, REVASCAT and ESCAPE (excluding those in whom a device other than Solitaire was used first). Panel A) overall results B) comparing age dichotomized at 70 years C) comparing age dichotomized at 80 years D) comparing those who did or did not receive intravenous alteplase prior to endovascular stent thrombectomy.

A) OVERALL

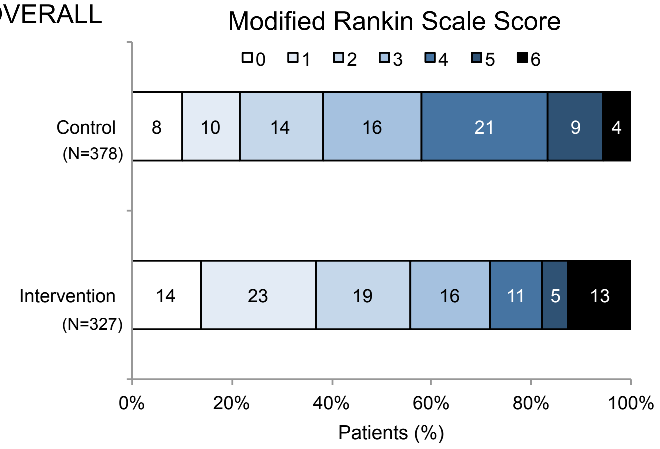

B) Age <70 versus ≥70

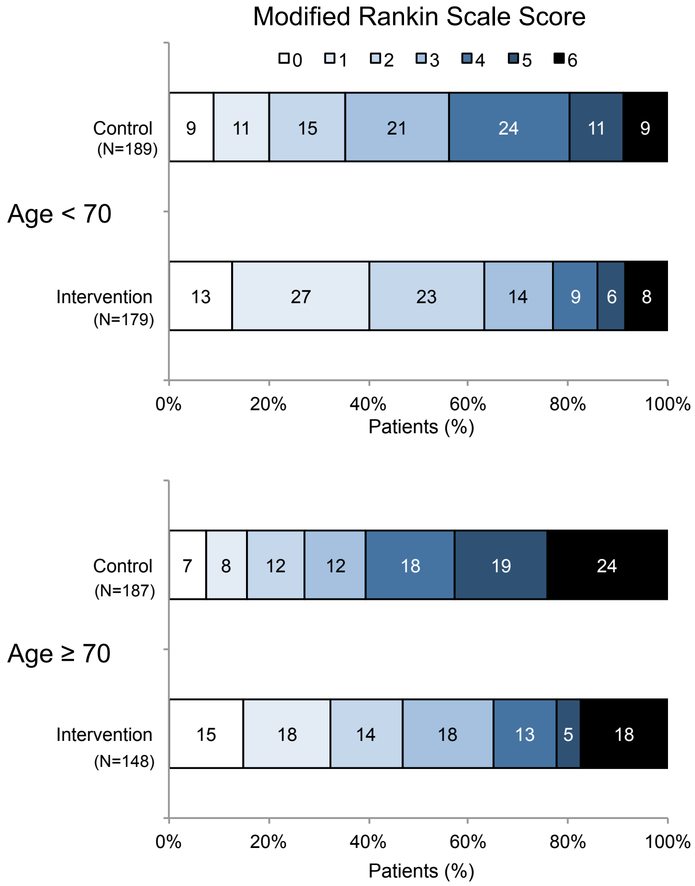

C) Age <80 versus ≥80

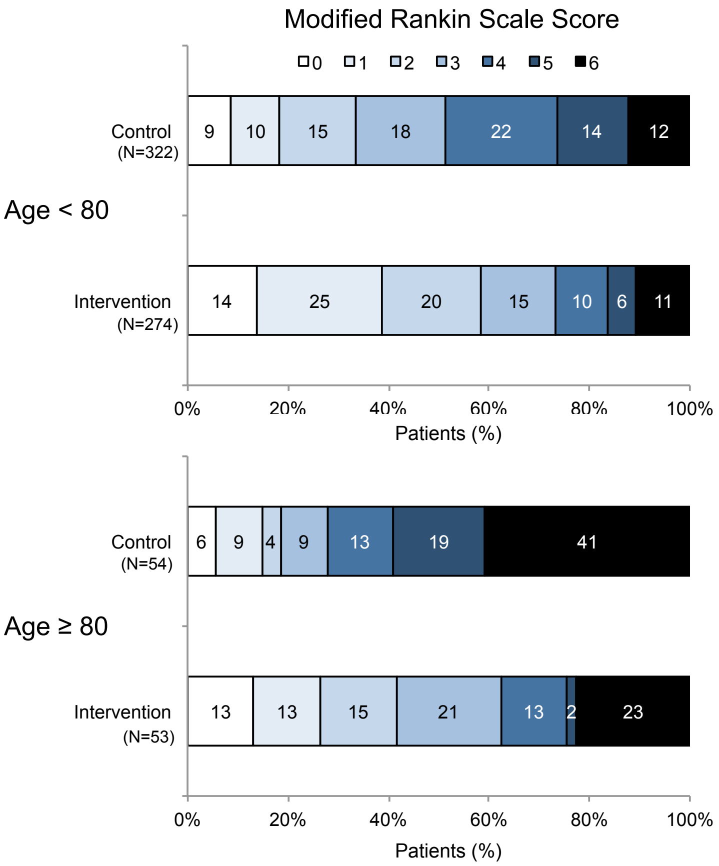

D) Alteplase vs no alteplase treatment

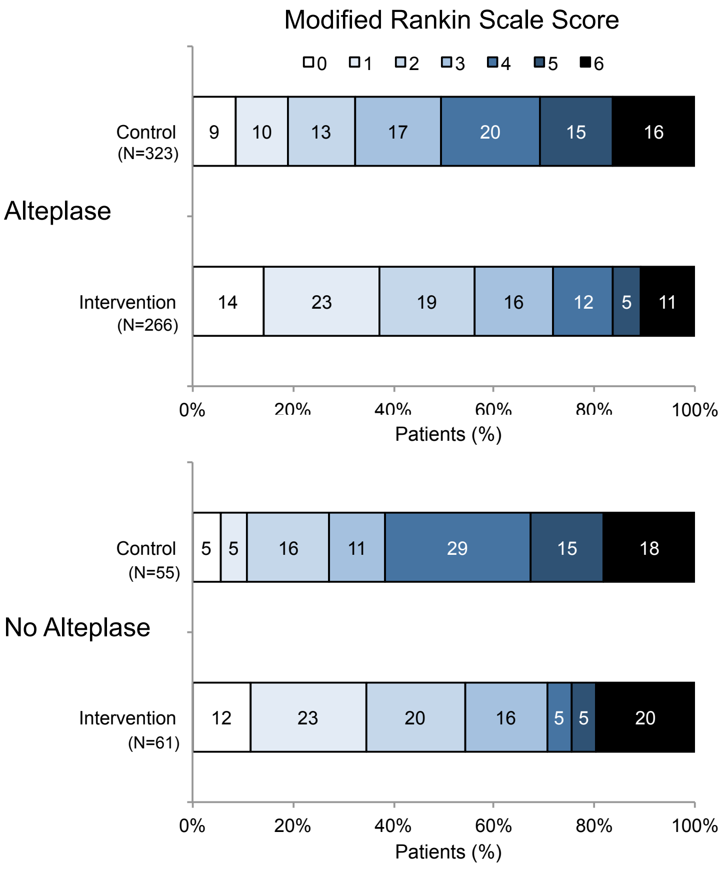

Figure II – Distribution of modified Rankin scores at 90 days in the sensitivity analysis 2: SWIFT PRIME, EXTEND-IA and REVASCAT (Solitaire-only trials). Panel A) overall results B) comparing age dichotomized at 70 years C) comparing age dichotomized at 80 years D) comparing those who did or did not receive intravenous alteplase prior to endovascular thrombectomy.

A) OVERALL

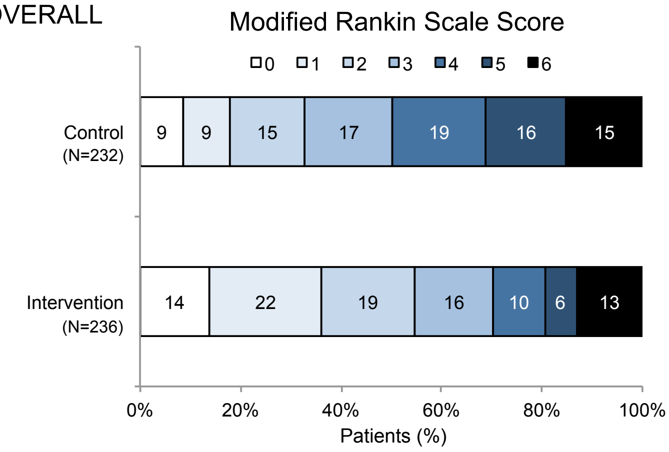

B) Age <70 versus ≥70

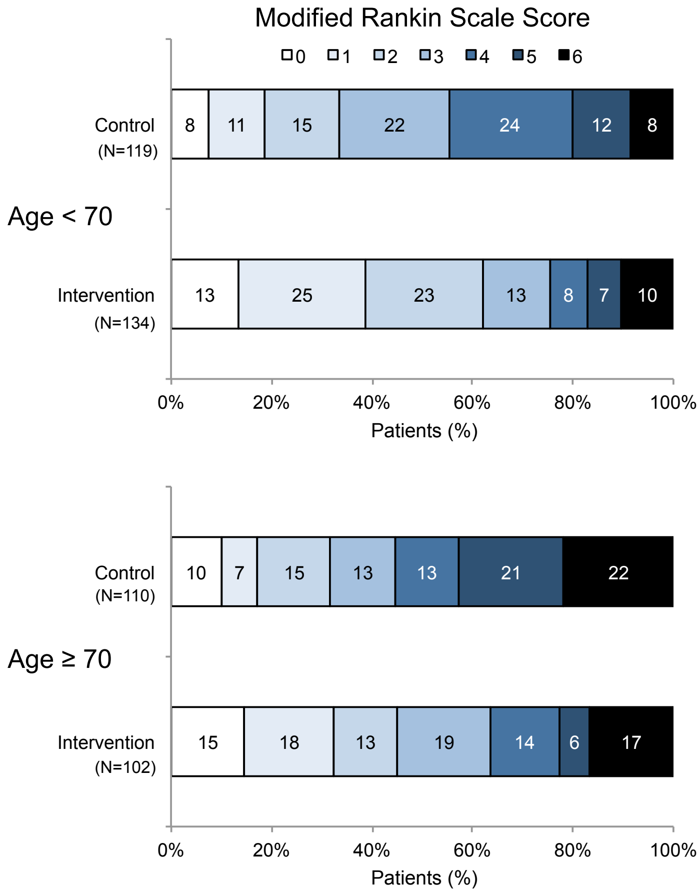

C) Age <80 versus ≥80

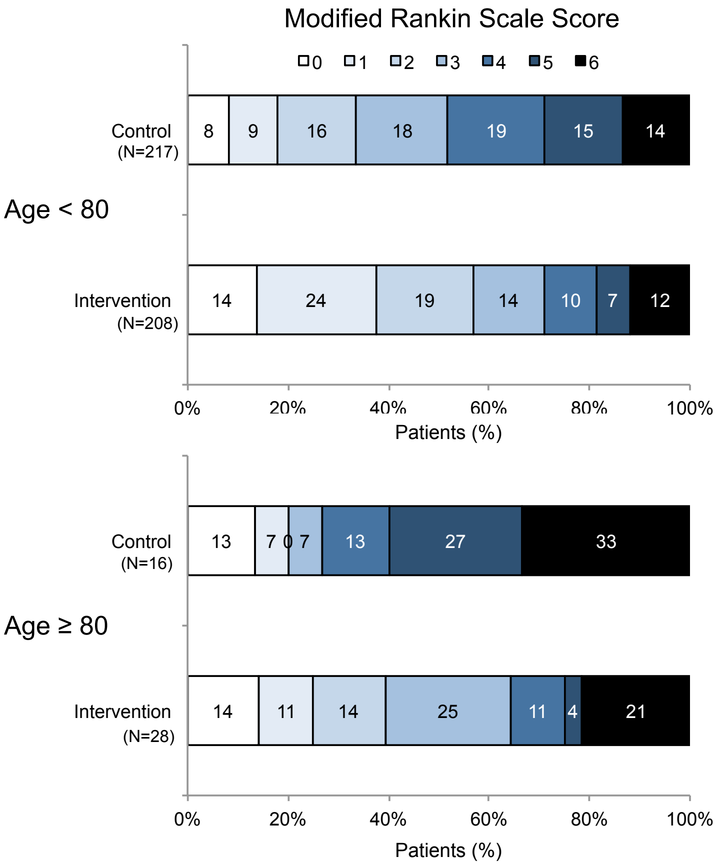

D) Alteplase vs no alteplase treatment

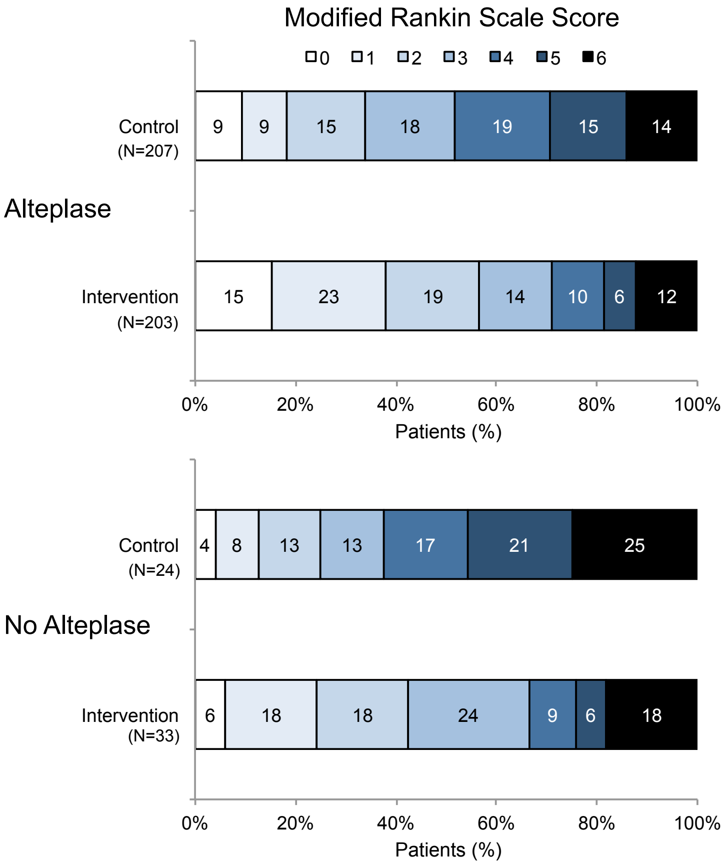

Figure III – Treatment effect in pre-defined subgroups (Forest plot) for sensitivity analysis 1: SWIFT PRIME, EXTEND-IA, REVASCAT and ESCAPE (excluding those in whom a device other than Solitaire was used first). Odds ratios with 95% confidence intervals, analyses adjusted for age, sex, baseline stroke severity, site of occlusion, intravenous alteplase treatment, ASPECTS score, and time from onset to randomization).

	Control n=386	Intervention n=327	Odds ratio (95% CI)	Interaction p-value
Age				
<70y	191	179	2.61 (1.77, 3.87)	0.34
≥70y	192	148	3.27 (1.99, 5.36)	
<80y	328	274	2.60 (1.91, 3.54)	0.09
≥80y	55	53	4.75 (1.95, 11.58)	
Sex				
Male	193	156	2.89 (1.92, 4.34)	0.77
Female	191	171	2.76 (1.86, 4.09)	
NIHSS				
0-15	155	137	3.26 (2.06, 5.14)	0.98
16-20	150	126	3.16 (1.99, 5.00)	
>20	76	62	3.67 (1.66, 8.12)	
Site of vessel occlusion				
ICA	66	52	6.80 (3.12, 14.81)	0.09
M1 MCA	287	237	2.53 (1.83, 3.49)	
M2 MCA	23	30	1.85 (0.57, 6.05)	
Tandem occlusion				
Tandem	41	33	6.04 (2.29, 15.90)	0.40
No Tandem	345	294	2.63 (1.95, 3.56)	
ASPECTS				
0-5	31	20	0.88 (0.24, 3.27)	**0.03**
6-8	152	138	2.55 (1.63, 4.00)	
9-10	197	160	3.40 (2.27, 5.08)	
IV thrombolysis				
Alteplase	327	266	2.65 (1.86, 3.77)	0.62
No alteplase	59	61	3.86 (1.81, 8.21)	
Onset to randomization time				
<5h	308	269	2.95 (2.16, 4.03)	0.50
>5h	75	58	2.18 (1.06, 4.49)	

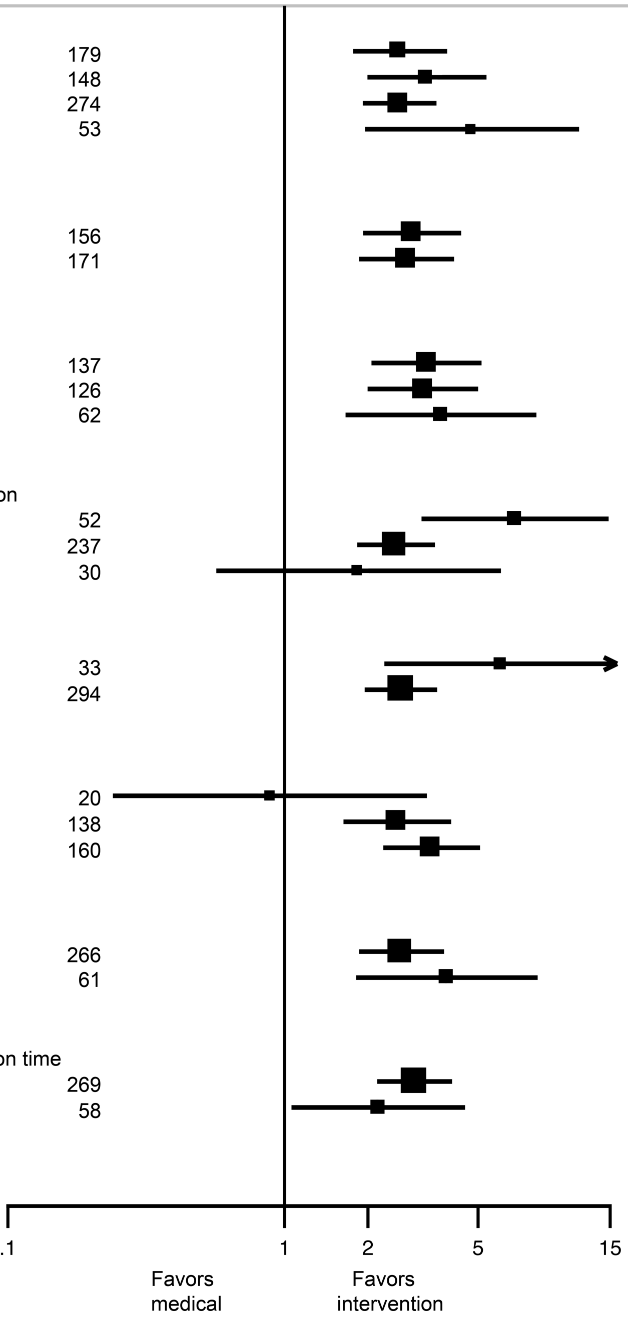

Figure IV – Treatment effect in pre-defined subgroups (Forest plot) for sensitivity analysis 2: SWIFT PRIME, EXTEND-IA and REVASCAT. Odds ratios with 95% confidence intervals, analyses adjusted for age, sex, baseline stroke severity, site of occlusion, intravenous alteplase treatment, ASPECTS score, and time from onset to randomization).
(Solitaire-only trials)

	Control n=236	Intervention n=236	Odds ratio (95% CI)	Interaction p-value
Age				
<70y	119	134	2.57 (1.60, 4.15)	0.78
≥70y	114	102	2.80 (1.59, 4.93)	
<80y	217	208	2.45 (1.70, 3.52)	0.24
≥80y	16	28	3.28 (0.46, 23.16)	
Sex				
Female	112	120	2.40 (1.47, 3.93)	0.77
Male	122	116	2.60 (1.59, 4.23)	
NIHSS				
0-15	97	90	2.67 (1.51, 4.72)	0.95
16-20	91	97	2.44 (1.34, 4.45)	
>20	44	49	3.60 (1.34, 9.68)	
Site of vessel occlusion				
ICA	27	28	10.71 (3.15, 36.44)	0.17
M1 MCA	182	174	2.18 (1.47, 3.22)	
M2 MCA	20	27	2.19 (0.56, 8.57)	
Tandem oclusion				
Tandem	22	22	6.44 (1.65, 25.23)	0.23
No Tandem	214	214	2.33 (0.85, 6.34)	
ASPECTS				
0-5	25	17	0.55 (0.12, 2.44)	0.22
6-8	98	103	2.82 (1.65, 4.81)	
9-10	107	110	2.67 (1.61, 4.43)	
IV thrombolysis				
Alteplase	209	203	2.35 (1.51, 3.66)	0.77
No alteplase	27	33	4.62 (1.41, 15.12)	
Onset to randomization time				
<5h	192	200	2.59 (1.78, 3.76)	0.39
>5h	41	36	1.86 (0.70, 4.96)	

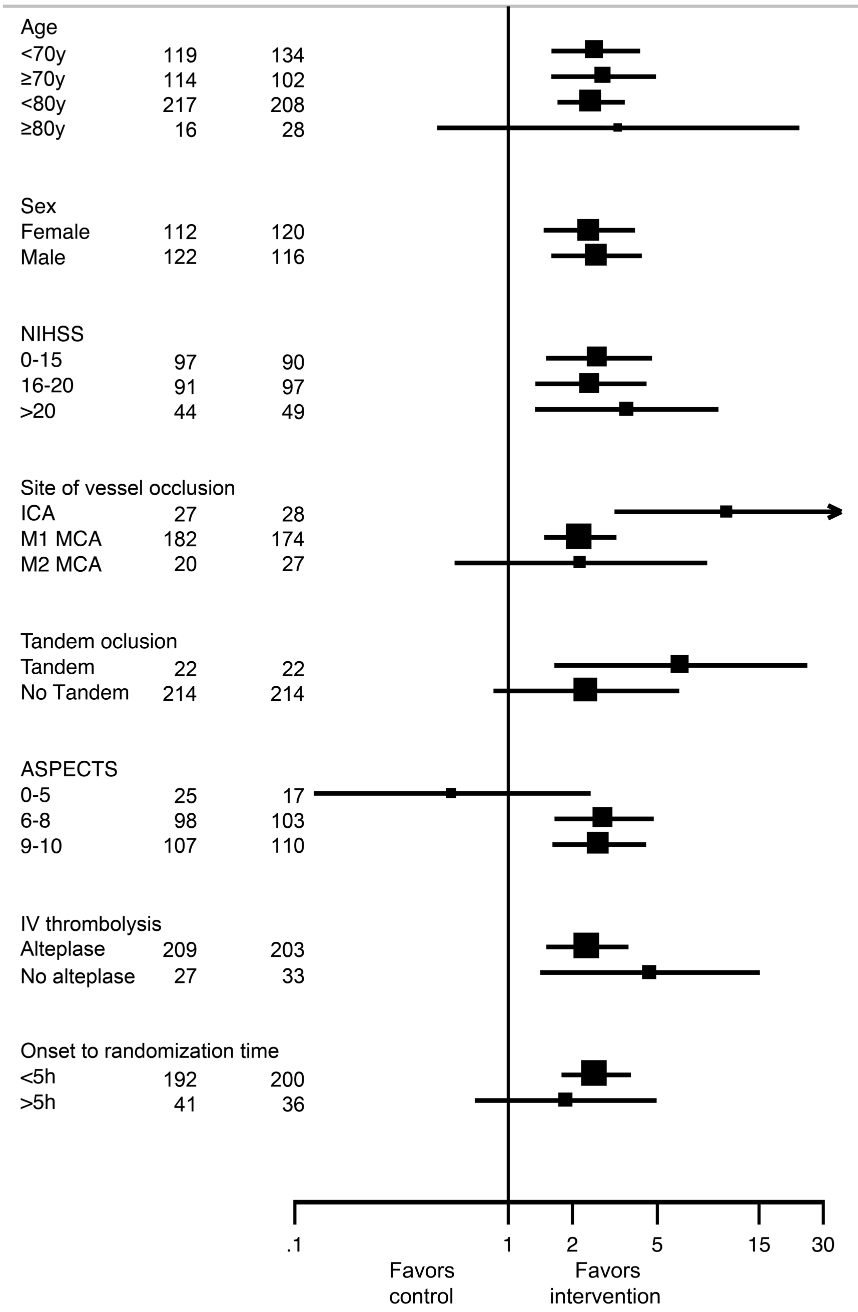

STATISTICAL ANALYSIS PLAN FOR THE INTEGRATION OF SAFETY AND EFFICACY DATA

PRODUCT UNDER INVESTIGATION:

Solitaire™ FR Revascularization Device

CLINICAL STUDIES FOR INTEGRATION:

EXTEND-IA: EXTENDING THE TIME FOR THROMBOLYSIS IN EMERGENCY NEUROLOGICAL DEFICITS – INTRA-ARTERIAL

ESCAPE: ENDOVASCULAR TREATMENT FOR SMALL CORE ANTERIOR CIRCULATION PROXIMAL OCCLUSION WITH EMPHASIS ON MINIMIZING CT TO RECANALIZATION TIMES

REVASCAT: RANDOMIZED TRIAL OF REVASCULARIZATION WITH SOLITAIRE FR® DEVICE VERSUS BEST MEDICAL THERAPY IN THE TREATMENT OF ACUTE STROKE DUE TO ANTERIOR CIRCULATION LARGE VESSEL OCCLUSION PRESENTING WITHIN 8 HOURS OF SYMPTOM ONSET

SWIFT PRIME: SOLITAIRE™ FR WITH THE INTENTION FOR THROMBECTOMY AS PRIMARY ENDOVASCULAR TREATMENT FOR ACUTE ISCHEMIC STROKE

Statistical Method and Analyses to Support the SEER working group (SWIFT PRIME, ESCAPE, EXTEND IA, REVASCAT)

DATE AND VERSION

November 1, 2015 (Version 1.0)

CONFIDENTIAL

CONFIDENTIALITY:
The information in this document is confidential and is the property of the SEER Working Group. The information may be provided only to Investigators, sub-Investigators, and appropriate trial personnel, members of the Institutional Review Board/Institutional Ethics Committee (IRB) and personnel of the authorities to whom it is submitted. This Statistical Analysis Plan may not be photocopied or discussed with third parties not involved in the trial without the prior written authorization from the SEER Working Group.

1. SIGNATURE PAGE

This document has been prepared by:

Biostatistician	Signature	Date (d/m/y)
Bruce C. Stouch, Ph.D.	Signature	Date

This document has been reviewed and accepted by:

SEER MEMBERS	Signature	Date (d/m/y)
Dr Michael Hill (ESCAPE)	Signature	Date
Dr Andrew Demchuk (ESCAPE)	Signature	Date
Dr Bruce Campbell (EXTEND-IA)	Signature	Date
Dr Peter Mitchell (EXTEND-IA)	Signature	Date
Dr. Tudor Jovin (REVASCAT)		
Antonio Davalos (REVASCAT)		
Dr. Jeff Saver (SWIFT PRIME)		

CONFIDENTIAL

TABLE OF CONTENTS

1. SIGNATURE PAGE 2

2. LIST OF ABBREVIATIONS AND DEFINITIONS 4

3. INTRODUCTION 8

3.1. Study Design and Enrollment for EXTEND-IA, ESCAPE, REVASCAT AND SWIFT PRIME 8

3.2. Selection of the computational Model for Analysis 12

4. ANALYSIS POPULATIONS 13

5. BASELINE INFORMATION 14

5.1. Evaluation and Integration of the Baseline Demographic Data for EXTEND-IA, ESCAPE, REVASCAT AND SWIFT PRIME 14

5.2. Evaluation and Integration of the Baseline Physical Characteristic Data for EXTEND-IA, ESCAPE, REVASCAT AND SWIFT PRIME 15

6. TPA AND MECHANICAL THROMBECTOMY INFORMATION 16

6.1. Evaluation of the Time to Specific Events 16

7. SAFETY INFORMATION 17

7.1. Assessment of the Safety Data for EXTEND-IA, ESCAPE, REVASCAT AND SWIFT PRIME 17

8. EFFICACY AND SAFETY INFORMATION 19

8.1. Evaluation of the 90-Day mRS Data for EXTEND-IA, ESCAPE, REVASCAT AND SWIFT PRIME 19

8.2. Evaluation of the 90-Day Mortality Data for EXTEND-IA, ESCAPE, REVASCAT AND SWIFT PRIME 20

8.3. Evaluation of Functional Independence at 90-Days for EXTEND-IA, ESCAPE, REVASCAT AND SWIFT PRIME 21

8.4. Evaluation of the Changes from Baseline in NIHSS at 90-Days for EXTEND-IA, ESCAPE, REVASCAT AND SWIFT PRIME 21

8.5. Evaluation of the effect of time from onset to reperfusion in the patents who achieved successful endovascular revascularization 21

CONFIDENTIAL

2. LIST OF ABBREVIATIONS AND DEFINITIONS

Abbreviation	Abbreviated Term	Definition
AAE	Anticipated Adverse Event	Any decline away from the patient's baseline health, whether related to the investigational device, the procedure or the disease that is predefined in the Investigational Plan, CRFs, and Instructions For Use.
ADE	Adverse Device Effect	Any untoward and unintended response to a medical device including insufficiencies or inadequacies in instructions for use or deployment of the device.
AE	Adverse Event	Any decline away from the patient's baseline health. Any decline from the patient's pre-treatment condition that occurs during the course of the clinical Study, after study enrollment, whether related to the investigational device, the procedure or the disease. Treatment includes all investigative or commercially approved products administered according to the Investigational Plan.
AICH	Asymptomatic Intracranial Hemorrhage	Any intracranial hemorrhage within 24 hours not meeting the criteria for symptomatic intracranial hemorrhage.
AIS	Acute Ischemic Stroke	Focal symptoms due to cerebral infarction from an arterial occlusion.
CEC	Clinical Events Committee	Independent committee responsible for the review and validation of all complications that occur over the course of the Study.
DICOM	Digital Imaging and Communications in Medicine	The standard foundation for imaging and image management; A global information-technology standard designed to ensure the interoperability of systems used to produce, store, display, process, send, retrieve, query, or print medical images and derived structured documents.
DSMB	Data Safety and Monitoring Board	An independent data monitoring committee, established by the sponsor, to assess at intervals, the progress of a clinical trial, the safety data and the critical efficacy endpoints, and to recommend to the sponsor whether to continue, modify or stop a trial.
DWI	Diffusion Weighted Imaging	Imaging obtained using magnetic resonance sequences that measure diffusion properties of water within tissue.
eCRF	Electronic Case Report Form	An electronic document designed to record all of the protocol requested information to be reported to the sponsor on each study patient. eCRFs are "living documents" in the respect that new information on the patient is continually gathered throughout the study.
FR	Flow Restoration	Restore flow through a vessel that is occluded by blood clot.
GCP	Good Clinical Practice	The regulations enforced in the US by the FDA's bioresearch monitoring program for medical devices, consisting of 21 CFR 812, -50 and -56. The GCP requirements are also stated in "Guidance for Industry, E6 Good Clinical Practice: Consolidated Guidance, ICH, April 1996: A standard for the design, conduct, performance, monitoring, auditing, recording, analyses, and reporting of clinical trials that provides assurance that the data and reported are credible and accurate, and that the rights, integrity and confidentiality of trial patients are protected.

CONFIDENTIAL

Abbreviation	Abbreviated Term	Definition
HIPAA	Health Insurance Portability and Accountability Act	Health Insurance Portability and Accountability Act of 1996. Title II of the Act, "Administrative Simplification" refers in large part to federal privacy rules that require health care providers and others to obtain written authorization from patients or their legally authorized representatives before using or disclosing their "Protected Health Information" (PHI) for any purposes other than treatment, billing, quality assurance and education.
ICF	Informed Consent Form	The written, signed and dated document that provides objective evidence of the process by which a patient voluntarily confirms his or her willingness to participate in a particular study, after having been informed of all aspects of the trial that are relevant to the patient's decision to participate (21 CFR 50).
ICH	International Conference for Harmonization	An Organization whose main purpose is to achieve greater harmonization to ensure that safe, effective, and high quality medicines are developed and registered in the most resource-efficient manner.
IDE	Investigational Device Exemption	An approved IDE permits a device that would otherwise be required to comply with a performance standard or would require a premarket approval to be shipped lawfully for the purpose of conducting investigations of that device (21 CFR 812).
I/E	Inclusion/Exclusio n Criteria	A list of conditions that would include or exclude a patient from enrolling/participating in a clinical study as outlined in the study protocol.
IEC	Independent Ethics Committee	An independent body, constituted of medical/scientific professionals and nonmedical/nonscientific members, whose responsibility it is to ensure the protection of the rights, safety and well-being of human patients involved in a trial and to provide public assurance of that protection, by, among other things, reviewing and approving/providing favorable opinion on the trial protocol, the suitability of the investigator(s), facilities and the methods and material to be used in obtaining and documenting informed consent of the trial patients.
INR	International Normalized Ratio	Ratio that measures the time it takes for blood to clot and compares it to a reference normal value.
IP	Investigational Plan	The clinical protocol and associated documents whose required composition is described in 21 CFR 812.25.
IRB	Institutional Review Board	Any board, committee, or other group formally designated by an institution to review biomedical research involving patients and established, operated and functioning in conformance with 21 CFR 56.
ISO	International Organization for Standardization	International standard-setting body composed of representatives from various national standards organizations.
ITT	Intent-to-Treat	The ITT population includes all patients with data for a given endpoint and are assessed according to randomized assignment regardless of the treatment actually received
IV t-PA	Intravenous Tissue Plasminogen Activator	Medical treatment of myocardial infarction with ST-elevation (STEMI), acute ischemic stroke (AIS), acute massive pulmonary embolism, and central venous access devices (CVAD) administered intravenously. t-PA is an enzyme (serine protease) found in endothelial cells that line the blood vessels that converts plasminogen into plasmin, an enzyme responsible for blood clot breakdown.
IVRS	Interactive Voice Response System	Accessed by telephone, it is a system that randomly assigns the patient to a treatment arm based on the pre-determined randomization algorithm.
IWRS	Interactive Web Response System	Accessed by internet, it is a system that randomly assigns the patient to a treatment arm based on the pre-determined randomization algorithm.

CONFIDENTIAL

Abbreviation	Abbreviated Term	Definition
MedDRA	Medical Dictionary for Regulatory Activities	Standardized medical terminology developed by ICH to facilitate sharing of regulatory information internationally for medical products used by humans. It is used for registration, documentation and safety monitoring of medical products both before and after a product has been authorized for sale. Products covered by the scope of MedDRA include pharmaceuticals, vaccines and drug-device combination products.
mRS	Modified Rankin Score	Scale for measuring the degree of disability or dependence in the daily activities of people who have suffered a stroke.<br>0 No symptoms at all<br>1 No significant disability despite symptoms; able to carry out all usual duties and activities<br>2 Slight disability; unable to carry out all previous activities, but able to look after own affairs without assistance<br>3 Moderate disability; requiring some help, but able to walk without assistance<br>4 Moderately severe disability; unable to walk without assistance and unable to attend to own bodily needs without assistance<br>5 Severe disability; bedridden, incontinent and requiring constant nursing care and attention<br>6 Dead
NIHSS	National Institute of Health Stroke Scale	Method for quantifying neurologic deficits developed by the National Institutes of Health. It is used to assess the severity of a stroke.
OC/RDC	ORACLE Clinical/ Remote Data Capture	EDC system that will be deployed to support data collection for this study.
PTT	Partial Thromboplastin Time	Measure of how long it takes for blood to clot. This test is used to determine if a patient has bleeding or clotting problems.
PWI	Perfusion weighted imaging	Imaging obtained using contrast that measures the brain perfusion including vascular transit time, cerebral blood volume, and cerebral blood flow.
QALY	Quality-adjusted life year	A measure that takes into account both the quantity and quality of life generated by healthcare interventions. It is the arithmetic product of life expectancy and a measure of the quality of the remaining life-years
RAPID	RApid processing of PerfusIon and Diffusion	A system that computes quantitative perfusion maps (cerebral blood volume, CBV; cerebral blood flow, CBF; mean transit time, MTT; and the time until the residue function reaches its peak, T(max)) using deconvolution of tissue and arterial signals.
SAE	Serious Adverse Event	Adverse Event that led to death or serious deterioration in the health of a patient that resulted in a life threatening illness or injury, permanent impairment of a body structure or a body function, hospitalisation or prolongation of existing hospitalisation, medical or surgical intervention to prevent permanent impairment to a body structure or a body function or is a congenital anomaly/birth defect.

CONFIDENTIAL

Abbreviation	Abbreviated Term	Definition
SICH	Symptomatic Intracranial Hemorrhage	Any PH1, PH2, RIH, SAH, or IVH associated with a 4 points or more worsening on the NIHSS within 24 hrs.<br>PH1: Hematoma within ischemic field with some mild space-occupying effect but involving ≤ 30% of the infarcted area.<br>PH2: Hematoma within ischemic field with space-occupying effect involving >30% of the infarcted area<br>RIH: Any intraparenchymal hemorrhage remote from the ischemic field<br>IVH: Intraventricular hemorrhage<br>SAH: Subarachnoid hemorrhage
TICI	Thrombolysis in Cerebral Infarction Perfusion Categories	0 = No perfusion. No antegrade flow beyond the point of occlusion.<br>1 = Perfusion past the initial obstruction but limited distal branch filling with little or slow distal perfusion<br>2A = Perfusion of less than half of the vascular distribution of the occluded artery (eg, filling and perfusion through 1 M2 division)<br>2B = Perfusion of half or greater of the vascular distribution of the occluded artery (eg, filling and perfusion through 2 or more M2 divisions)<br>3 = Full perfusion with filling of all distal branches
UADE	Unanticipated Adverse Device Effect	Any serious adverse effect on health or safety or any life-threatening problem or death caused by, or associated with, a device, if that effect, problem, or death was not previously identified in nature, severity or degree of incidence in the Investigational Plan or application (including a supplementary plan or application), or any other unanticipated serious problem associated with a device that relates to the rights, safety, or welfare of patients.
ULN	Upper Limit of Normal	Upper limit within a particular range.

CONFIDENTIAL

3. INTRODUCTION

This Statistical Analysis Plan (SAP) describes the statistical methods to be used for the analysis of the integrated safety and efficacy data from 4 randomized parallel-design studies where the objective was to treat patients presenting with an acute ischemic stroke. This plan has 5 elements:

- Comparison of the baseline patient-level data across the 4 studies,
- statistical analyses to evaluate safety and efficacy across the 4 studies using the analysis dataset rules from each of the individual studies,
- pooling the statistical evidence across the 4 randomized studies using the analysis dataset rules from each of the individual studies,
- pooling the patient-level data across the 4 randomized studies, generating an integrated database with a common set of analysis dataset rules
- statistical analyses to evaluate safety and efficacy across the 4 studies using the integrated database with a common set of analysis dataset rules

The tabulations and information from this SAP will provide an unbiased examination of the safety and efficacy of mechanical embolectomy relative to medical management alone. The integrated database will also allow examination of the safety and efficacy of mechanical embolectomy with the Solitaire FR device.

These analyses will be presented to the SEER committee for review, publication, and presentation. The approach used for the integration outlined in this document will also generate the requisite information to support the generation of an integrated Clinical Study Report (CSR), including the detailed descriptions of the statistical methodologies to be applied.

The structure and content of this plan provides sufficient detail to meet the requirements identified by the FDA and International Conference on Harmonization of Technical Requirements for medical devices, as well as the Registration of Pharmaceuticals for Human Use (ICH): Guidance on Statistical Principles in Clinical Trials.

3.1. Study Design and Enrollment for EXTEND-IA, ESCAPE, REVASCAT AND SWIFT PRIME

The clinical trial designs and control groups were similar across the 4 studies. The source of the summarized information from each study, along with the information regarding recruitment, was ClinicalTrials.gov.

ESCAPE was a multi-center, randomized, single blind (Outcomes Assessor) parallel design study open to patients ≥18 years of age enrolled within 12 hours of last being seen normal with a baseline NIHSS > 5 at the time of randomization. Several devices were permitted to be used, with the Solitaire stent retriever used in the majority. On the advice of the DSMB, trial recruitment was halted for efficacy and the study was terminated.

EXTEND-IA was a multi-center, randomized, single blind (Outcomes Assessor) parallel design study open to patients ≥18 years of age enrolled within 4.5 hours of stroke onset. Only the

CONFIDENTIAL

Solitaire stent retriever device was used. On the advice of the DSMB, trial recruitment was halted for efficacy and the study was terminated.

REVASCAT was a multi-center, randomized, single blind (Outcomes Assessor) parallel design study open to patients 18 to 85 years of age presenting within 8 hours of symptom onset consistent with an acute ischemic stroke. Only the Solitaire stent retriever device was used. On the advice of the DSMB, trial recruitment was halted for efficacy and the study was terminated.

SWIFT PRIME was a multi-center, randomized, single blind (Outcomes Assessor) parallel design study open to patients 18-85 years of age. Eligible patients were treated within 6 hours of the onset of stroke symptoms and within 1.5 hours from CTA or MRA to groin puncture and had a baseline NIHSS ≥ 8 and < 30 at the time of randomization. Only the Solitaire stent retriever device was used. This study was completed in January 2015.

Collectively, the primary objective of all 4 studies was the same: to evaluate the hypothesis that mechanical embolectomy is superior to medical management alone in achieving a favorable outcome based on the distribution of the modified Rankin Scale at 90 days in patients presenting with an acute large vessel ischemic stroke.

The methods for assigning patients to either mechanical embolectomy or medical management through randomization were also similar across the 4 studies regarding stratification.

For EXTEND-IA, patients were randomized to receive intra-arterial clot retrieval after IV tPA or IV tPA alone. Patients randomized to treatment were stratified for site of baseline arterial occlusion into one of three groups: Internal carotid artery (ICA), proximal middle cerebral artery (MCA –M1), or distal middle cerebral artery (MCA – M2).

For ESCAPE, patients were randomized to receive intra-arterial clot retrieval after IV tPA or IV tPA alone. The randomization was stratified based on age, baseline stroke severity (NIHSS), initial arterial lesion location, ASPECTS score, and site.

For REVASCAT, patients were randomly assigned in an equal ratio to either mechanical embolectomy or medical management, stratifying on age (≤70 or >70 years), baseline NIHSS (<17 verses >=17), therapeutic window (≤4.5 or >4.5 hours), investigational site and occlusion site (intracranial ICA or M1).

For SWIFT PRIME, patients were randomized to IV tPA alone or IV tPA plus Solitaire The randomization ratio for mechanical embolectomy or medical management was 1:1 and balanced within investigational sites and by baseline NIHSS severity (<= 17 versus >17), age (<70 years versus >=70 years at the time of randomization) and occlusion location (M1 versus all other).

The time to treatment was an important factor for inclusion in each of the 4 studies. For EXTEND-IA, patients were to be enrolled within 6 hours of stroke onset. For ESCAPE, the time to treatment had to be within 12 hours of stroke symptoms, endovascular treatment (groin puncture) within 60 minutes, target CT to first recanalization of 90 minutes. For REVASCAT, the time to treatment had to be within 8 hours of symptom onset. For SWIFT PRIME, the time to treatment had to be within 6 hours of onset of stroke symptoms and within 1.5 hours (90 minutes) from CTA or MRA to groin puncture.

CONFIDENTIAL

Time Points for Evaluation Across the 4 Studies

The schedule of assessment for each of the 4 randomized studies only fully aligned for the baseline / pre-procedure and 90 day evaluation. The post-randomization evaluations were similar in terms of timing; however, the time windows for evaluation did not completely align. Specifically, the first post-procedure assessment in SWIFT PRIME had a 27 hour +/- 6 hour window (21 to 33 hours post procedure). ESCAPE defines the time point simply as 24 hours or 1 day; it is indeterminate what the exact window was; this will be quantified based on the actual data. REVASCAT had a 22 to 36 hour time window. For integration purposes, all data collected at the ~24 hour post-randomization evaluation will be integrated for the Level 1 presentation and the distributions will be compared across the 4 randomized studies. For the Level 2 presentation of the safety and efficacy results, only evaluations recorded within 21 to 36 hours of randomization will be considered.

Multiple observations for a patient within a time window

If a patient has multiple observations within a time window for the -24 hour post randomization evaluation, the value recorded closest to 24 hours will be used.

Retaining Clinical Center or Site in the Model for Analysis

In each of the 4 multicenter studies, the randomization scheme was balanced intra-center. Within each center, patients were stratified relative to a number of factors. The timing of individual examinations and the time windows are presented below.

Evaluations	Pre-Rx	Rx	2-8 H	D 1	D 2	D 3	D 5	D 7-10	D 30	D 90
ESCAPE	•	•	•	•	•		•		•	•
EXTEND-IA	•	•		•		•				•
REVASCAT	•	•		•			•			•
SWIFT PRIME	•	•		•				•	•	•

Time Windows	D 1	D 2	D 3	D 5	D 7-10	D 30	D 90
ESCAPE	18H-30H	42H to 54H		D4 to D6		D25 to D35	D83 to D97
EXTEND-IA	18H-30H		D2 to D4				D83 to D97
REVASCAT	22H to 36H			D3 to D7			D76 to D104
SWIFT PRIME	21H to 33H				D7 to D10	D23 to D37	D75 to D105

CONFIDENTIAL

MRS	Pre-Rx	Rx	2-8 H	D 1	D 2	D 3	D 5	D 7-10	D 30	D 90
ESCAPE				• 24+/-6 hours post-random.					•	•
EXTEND-IA	•									•
REVASCAT	•						•			•
SWIFT PRIME	•			• 27+/-6 hours post-random.				•	•	•

NIHSS	Pre-Rx	Rx	2-8 H	D 1	D 2	D 3	D 5	D 7-10	D 30	D 90
ESCAPE	•	•		•			•		•	•
EXTEND-IA	•			•		•				•
REVASCAT	•			•			•			•
SWIFT PRIME	•			•				•	•	•

BARTHEL	Pre-Rx	Rx	2-8 H	D 1	D 2	D 3	D 5	D 7-10	D 30	D 90
ESCAPE										
EXTEND-IA										
REVASCAT										•
SWIFT PRIME								•		•

CONFIDENTIAL

3.2. Selection of the computational Model for Analysis

The selection of a computational model was based on our expectation about whether or not the studies shared a common effect size, and on our research goals in performing the analysis. The use of a fixed-effect model would be appropriate if we believed that the studies are functionally identical, and our goal is to compute the common effect size for the identified population, and not to generalize to other populations. Given the studies were all conducted independently and in different geographic locations, it would be unlikely that all the studies were functionally equivalent. The consensus opinion of the SEER working group was that these studies differed in ways that could impact the outcomes and therefore one should not assume a common effect size. Additionally, the goal of this analysis is to generalize to a range of scenarios. Therefore, if one did make the argument that all the studies used an identical, narrowly defined population, then it would not be possible to extrapolate from this population to others, and the utility of the analysis would be severely limited. We acknowledge the number of studies being analyzed is small and the estimate of the between-studies variance may lack precision.

In summary, a fixed-effect meta-analysis estimates a single effect that is assumed to be common to every study, while a random-effects meta-analysis estimates the mean of a distribution of effects. Study weights are more balanced under the random-effects model than under the fixed-effect model. Large studies are assigned less relative weight and small studies are assigned more relative weight as compared with the fixed-effect model. The standard error of the summary effect and the confidence intervals for the summary effect are wider under the random-effects model than under the fixed-effect model. The selection of a model must be based solely on the question of which model fits the distribution of effect sizes, and takes into account the relevant sources of error; for this reason, we believe the application of a more conservative random effects model is justified.

CONFIDENTIAL

4. ANALYSIS POPULATIONS

The following populations will be considered for the integration and presentation of the data:

Enrolled Population

The ***Enrolled Population*** includes all consented patients in each of the 4 studies who were randomized, independent of the administration of tPA or the outcome of the procedure. The randomized groups for comparison will be referred to as the ***Standard of Care Plus Thrombectomy*** and ***Standard of Care Alone***. In the analysis tables, the ***Standard of Care Plus Thrombectomy*** will be referred to as the ***Device Group***; ***Standard of Care Alone*** will be referred to as the ***Control Group***.

The primary analysis will be performed in the 3 trials that used only the Solitaire device (SWIFT PRIME, EXTEND-IA and REVASCAT. Two sensitivity analyses will be performed including additional data from the ESCAPE trial 1) including only those patients from the endovascular arm in which the first device actually used was Solitaire or would have been used had a target clot been still present and accessible (Solitaire intention to treat analysis), and 2) including all patients in the endovascular arm, whether or not Solitaire was used first (4 trial analysis).

Addendum 30-Dec-2015 – in response to journal reviewer's suggestion, the primary analysis was changed to be the full four trial dataset and the 3 trial analysis was instead presented as the second sensitivity analysis.

IV tPA Treated Within 3-Hours Population (in accordance with FDA label for alteplase)

The ***IV tPA Treated Within 3-Hours Population*** includes all consented patients in each of the 3 populations studied who were administered IV tPA within 3 hours of the stroke symptom onset.

CONFIDENTIAL

5. BASELINE INFORMATION

The demographic, physical characteristic, neurologic history and pre-procedure imaging data will be evaluated to assess the magnitude of the differences in the patients enrolled in the 4 studies. Probability values to evaluate the differences will be considered significant if the value is <0.05. The description presented below describes the method of summarization and comparison of the following factors:

- Age (years)
- Sex
- Race
- NIHSS (Baseline) Score
- Previously Diagnosed Atrial Fibrillation
- Hypertension
- Diabetes Mellitus
- Current or Past Tobacco Use
- Serum Glucose (mg/dL)
- Site of Vessel Occlusion
- Non-Contrast CT ASPECTS

5.1. Evaluation and Integration of the Baseline Demographic Data for EXTEND-IA, ESCAPE, REVASCAT AND SWIFT PRIME

All enrolled patients from the 4 studies will be summarized with respect to age at entry into the study, Sex, ethnicity, and race. Age will be calculated as [Date of Informed Consent – Date of Birth] / 365.25 rounded down to the nearest integer. Age will be reported in years (yr) and summarized using descriptive statistics: n, arithmetic mean, standard deviation, median, range (i.e., minimum and maximum values) and compared between the randomized treatment assignments using a mixed model specifying the distribution as continuous with study and study-by-treatment as random effects. Patients with missing data that cannot be resolved will not be included in the tabulation and excluded from the summary statistics. The number and percent of patients ≤70 and > 70 and ≤80 and >80 years of age at the time of enrollment will be presented by randomized treatment assignment and study, and independent of study using counts and percentages. The proportion of patients in each mutually-exclusive category will be compared between the randomized groups using a mixed model specifying the distribution as binomial with study and study-by-treatment as random effects. Results will also be generated for the IV tPA Treated Population (Tables 1a) and the IV tPA Treated Within 3-Hours Population (Tables 1b).

CONFIDENTIAL

5.2. Evaluation and Integration of the Baseline Physical Characteristic Data for EXTEND-IA, ESCAPE, REVASCAT AND SWIFT PRIME

All enrolled patients from the 4 studies will be presented in Table 2.0 entitled *Summary of Physical Characteristics Recorded at Baseline* (Enrolled Population). This table will summarize the physical characteristic data by study with respect to height (inches), weight (lbs.), BMI, and Pre-stroke mRS. Height, weight, BMI, and pre-stroke mRS will be summarized using descriptive statistics: n, arithmetic mean, standard deviation, median, range (*i.e.*, minimum and maximum values) and compared between the randomized treatment assignments using a mixed model specifying the distribution as continuous with study and study-by-treatment as random effects. Patients with missing data that cannot be resolved will not be included in the tabulation and excluded from the summary statistics.

CONFIDENTIAL

6. TPA AND MECHANICAL THROMBECTOMY INFORMATION

Patients will be classified as having received tPA (Yes or No) and if they underwent mechanical thrombectomy (Yes or No). Specific information regarding the time to administration of tPA and the thrombectomy procedure will also be used in the logistic and random-effect models.

6.1. Evaluation of the Time to Specific Events

- Time (minutes) from Stroke Onset to Hospital Arrival
- Treatment with Intravenous Alteplase
- Time (minutes) from Stroke Onset to Treatment with Intravenous Alteplase
- Time (minutes) from Hospital Arrival to Treatment with Intravenous Alteplase
- Time (minutes) from Treatment with Intravenous Alteplase to Randomization
- Time (minutes) from Stroke Onset to Arterial Access
- Time (minutes) from Hospital Arrival to Arterial Access
- Time (minutes) from Initial Imaging to Arterial Access
- Time (minutes) from Treatment with Intravenous Alteplase to Arterial Access
- Time (minutes) from Arterial Access to mTICI 2b/3 or Completion
- Time (minutes) from Stroke Onset to mTICI 2b/3 or Completion

Individual times will be summarized using descriptive statistics: n, arithmetic mean, standard deviation, median, range (*i.e.*, minimum and maximum values) and compared between the randomized treatment assignments using a mixed model specifying the distribution as continuous with study and study-by-treatment as random effects. Patients with missing data that cannot be resolved will not be included in the tabulation and excluded from the summary statistics.

CONFIDENTIAL

7. SAFETY INFORMATION

The assessment of safety will be based on adverse events reported within 90 days of randomization and the incidence of symptomatic ICH (intracranial hemorrhage) within 21 to 36 hours of randomization.

7.1. Assessment of the Safety Data for EXTEND-IA, ESCAPE, REVASCAT AND SWIFT PRIME

The incidence of symptomatic ICH will be defined in accordance with the definitions used in each trial.

Adverse events will be presented according to 3 time intervals: peri-procedural adverse events, adverse events reported within 30 days of randomization, and adverse events within 90 days of randomization. *Peri-procedural adverse events* are events that occur during the thrombectomy procedure

An adverse event is defined as any decline away from the patient's baseline health. Any decline from the patient's pre-treatment condition that occurs during the course of the clinical Study, after starting treatment, whether related to the investigational device, the procedure or the disease. A serious adverse event is an event that leads to death, fetal distress, fetal death or a congenital abnormality or birth defect, and serious deterioration in the health of a patient that resulted in a life-threatening illness or injury such as a permanent impairment of a body structure or a body function, requiring in-patient hospitalization or prolongation of existing hospitalization, results in medical or surgical intervention to prevent permanent impairment to a body structure or a body function. Treatment includes all investigative or commercially approved products administered according to the Investigational Plan from each of the 4 studies. The following information regarding each adverse event includes the date and time of onset and resolution (duration), intensity, whether it was serious, any required treatment or action taken, outcome, relationship to the investigational procedure or tPA, and whether the adverse event caused withdrawal from the study.

Mild: Patient is aware of event or symptom but event/symptom is easily tolerated.

Moderate: Patient experiences sufficient discomfort to interfere with or reduce their usual level of activity.

Severe: Significant impairment of functioning; patient is unable to carry out usual activities.

The relationship of the adverse events across the studies were defined using different scales.

For SWIFT PRIME, there were 9 coded categories:
1 =IV t-PA Related,
2=Device Related: Solitaire,
3=Device Related: Ancillary,
4=Device Related: Unknown,
5=Procedure/Treatment Related,
6=Study Disease Related,
7=Concomitant Disease,

CONFIDENTIAL

8=Unknown, and
9=Other.

For ESCAPE, there were 5 coded categories for relationship to the *investigational product*:
1=Not related,
2=Unlikely,
3=Possible,
4=Probable,
5=Definite

For ESCAPE, there were 2 categories for relationship to the endovascular procedure; yes or no.

For REVASCAT, *relationship* to the investigational device or procedure was collected using the following 5 categories:

1=Definitive
2=Probable
3=Possible
4=Conditional
5=Not related

For EXTEND-IA, *relationship* to the investigational device was collected using the following 5 categories:

1=Not related,
2=Unlikely,
3=Possible,
4=Probable,
5=Definite

CONFIDENTIAL

8. EFFICACY AND SAFETY INFORMATION

There are 7 outcomes that will be summarized using the integrated data from the 4 studies in the primary and two sensitivity analysis populations described above. The primary outcome is ordinal analysis of the mRS at 90 days. Secondary outcomes are functional independence (mRS ≤ 2 at 90 days), excellent functional outcome (mRS 0-1), the proportion of patients with a reduction in NIHSS between baseline and 21-36 hours of ≥8 points or reaching 0-1, death due to any cause at 90 days, symptomatic intracerebral hemorrhage and parenchymal hematoma.

8.1. Evaluation of the 90-Day mRS Data for EXTEND-IA, ESCAPE, REVASCAT AND SWIFT PRIME

The mRS is a well-accepted and defined scoring system for measuring the degree of disability or dependence in the daily activities of people who have suffered a stroke using a 0-6 multinomial ordinal scale:

0 No symptoms at all
1 No significant disability despite symptoms; able to carry out all usual duties and activities
2 Slight disability; unable to carry out all previous activities, but able to look after own affairs without assistance
3 Moderate disability; requiring some help, but able to walk without assistance
4 Moderately severe disability; unable to walk without assistance and unable to attend to own bodily needs without assistance
5 Severe disability; bedridden, incontinent and requiring constant nursing care and attention
6 Dead

The statistical hypothesis on Rankin shift is that the distribution of mRS in patients randomized to the treatment (Standard of Care plus Embolectomy) will be more favorable than the distribution in the control group (Standard of Care alone). For this purpose, the entire distribution 0 to 6 of mRS values will be considered except that categories 5 and 6 are collapsed into a single group.

The null and alternative hypotheses to be tested appear below:

$$H_o: \pi t = \pi c$$
$$H_a: \pi t < \pi c,$$

where πt is the central tendency of the mRS distribution at the 90-day follow-up visit in the treatment group, and πc is the corresponding value in the control group, with lower values indicating better outcomes.

A random-effects generalized linear model for independent data by maximum likelihood that allows for subject-specific (conditional) and population-averaged (marginal) inference (PROC GLIMMIX) will also be generated, incorporating predictive factors and stratification variables specified in the randomization as covariates: NIHSS recorded at baseline, age, sex, time to tPA,

occlusion location, time from onset to randomization and baseline ASPECTS score. The SAS code used to conduct the adjusted analysis is presented below.

```
proc glimmix data=MASTER_001 method=laplace;
  class SEX OCC_SITE TPA_YN TREAT_N STUDY;
  model MRSD90 = AGE_N SEX NIHSS_BL ASPECTS OCC_SITE TPA_YN TREAT_N
SYM_RAND_MIN / dist=multinomial link=cumlogit ddfm=residual solution cl or;
  random STUDY STUDY * TREAT_N / solution;
run;
```

The SAS code used to conduct the unadjusted analysis is presented below.

```
proc glimmix data=MASTER_001 method=laplace;
  class TREAT_N STUDY;
  model MRSD90 = TREAT_N / dist=multinomial link=cumlogit ddfm=residual solution cl or;
  random STUDY TREAT_N*STUDY / solution;
run;
```

The number needed to treat (NNT) to achieve an improvement by at least one level in the mRS will be calculated as the average of the algorithmic joint outcome table and the permutation method.

Separate sub-group analyses will be prepared to examine factors that may have influenced the 90-day mRS scores. Factors include: age (<70 years of age vs ≥70 years and <80 years of age vs ≥80 years), sex (male/female), stroke severity (≤15, 16-20, ≥21), site of intracranial vascular occlusion (ICA, M1, M2), presence of tandem cervical carotid occlusion (yes/no), extent of initial early infarct signs (ASPECTS 0-5, 6-8, 9-10), administration of alteplase (yes/no) and time from onset to randomization (<5h, ≥ 5h). Onset to randomization dichotomization at 5h was chosen to approximate the subgroup who could have endovascular treatment commenced within 6h of onset.

8.2. Evaluation of the 90-Day Mortality Data for EXTEND-IA, ESCAPE, REVASCAT AND SWIFT PRIME

The incidence of 90-day mortality will be compared across studies and between the randomized treatment groups.

CONFIDENTIAL

8.3. Evaluation of Functional Independence at 90-Days for EXTEND-IA, ESCAPE, REVASCAT AND SWIFT PRIME

Functional independence as defined by a modified Rankin Scale (mRS) score ≤ 2 at 90 days will be compared across the studies and between the randomized treatment groups. The SAS code used to conduct the adjusted analysis is presented below.

```
proc glimmix data=MASTER_001 METHOD=RSPL;
  class SEX OCC_SITE TPA_YN TREAT_N STUDY;
  model MRSD90=AGE_N SEX NIHSS_BL ASPECTS OCC_SITE TPA_YN
SYM_RAND_MIN TREAT_N / DIST=binary ddfm=residual link=logit cl or ODDSRATIO;
  random STUDY STUDY*TREAT_N / solution;
run;
```

The SAS code used to conduct the unadjusted analysis is presented below.

```
proc glimmix data=MASTER_001 METHOD=RSPL;
  class TREAT_N STUDY;
  model MRSD90 = TREAT_N / DIST=binary ddfm=residual link=logit cl or ODDSRATIO;
  random STUDY STUDY*TREAT_N / solution;
run;
```

8.4. Evaluation of the Changes from Baseline in NIHSS at 90-Days for EXTEND-IA, ESCAPE, REVASCAT AND SWIFT PRIME

The changes in the NIH Stroke Scale score from baseline to 21 to 36 hours after randomization will be compared among the studies and between the randomized treatment groups. Results will be summarized using descriptive statistics: n, arithmetic mean, standard deviation, median, range (i.e., minimum and maximum values) and compared between the randomized treatment assignments using a mixed model specifying the distribution as continuous with study and study-by-treatment as random effects. Patients with missing data that cannot be resolved will not be included in the tabulation and excluded from the summary statistics.

8.5. Evaluation of the effect of time from onset to reperfusion in the patents who achieved successful endovascular revascularization

A limited assessment of time as a predictor of outcome will be conducted in the intervention group. The adjusted probability of independent outcome in the intervention group that achieved mTICI 2b/3 reperfusion will be solved using an hierarchical generalized linear model with study as a grouping variable and onset-to-reperfusion time. Probabilities will be graphed as a function of time with the probability of independent outcome regressed against time using simple linear regression to produce an estimate of effect size for delay to treatment.

CONFIDENTIAL
